# Overcoming Photodynamic Therapy Resistance: GSH‐Responsive AIE Photosensitizer‐Hydroxycamptothecin Nanoliposomes for Extrahepatic Cholangiocarcinoma

**DOI:** 10.1002/adhm.71599

**Published:** 2026-08-13

**Authors:** Yong Qu, Tao Peng, Yating Zhang, Juanmei Cao, Birong Wang, Chuxing Chai, Zhiyuan Gao, Yiting Xu, Yifan Jin, Yuqing Wang, Zhuoxia Li, Xu Liu, Tianqi Zhao, Yufan Chen, Weimin Lv, Di Lu, Changzheng Huang, Dan Ding, Jinxiang Zhang, Min Li

**Affiliations:** ^1^ Department of Hepatobiliary Surgery Union Hospital Tongji Medical College Huazhong University of Science and Technology Wuhan China; ^2^ Department of Emergency Surgery Union Hospital Tongji Medical College Huazhong University of Science and Technology Wuhan China; ^3^ Department of Hepatobiliary Surgery The First People's Hospital of Jingzhou The First Affiliated Hospital of Yangtze University Jingzhou China; ^4^ Department of Hepatobiliary Surgery Division of Life Sciences and Medicine The First Affiliated Hospital of USTC University of Science and Technology of China Hefei China; ^5^ Department of Dermatology The First Affiliated Hospital of Shihezi University Shihezi China; ^6^ Department of Thyroid Gland and Breast Surgery Wuhan Fourth Hospital Wuhan China; ^7^ Frontiers Science Center For Cell Responses State Key Laboratory of Medicinal Chemical Biology Key Laboratory of Bioactive Materials and College of Life Sciences Ministry of Education Nankai University Tianjin China; ^8^ Central Laboratory Union Hospital Tongji Medical College Huazhong University of Science and Technology Wuhan China; ^9^ Hospital For Skin Diseases Institute of Dermatology Chinese Academy of Medical Sciences & Peking Union Medical College Nanjing China; ^10^ Department of Clinical Nutrition Union Hospital Tongji Medical College Huazhong University of Science and Technology Wuhan China; ^11^ Department of Dermatology Union Hospital Tongji Medical College Huazhong University of Science and Technology Wuhan China

**Keywords:** aggregation‐induced emission, chemotherapy, extrahepatic cholangiocarcinoma, hydroxycamptothecin, photodynamic therapy

## Abstract

Photodynamic therapy (PDT) has been shown to improve survival and quality of life in patients with unresectable extrahepatic cholangiocarcinoma. However, its therapeutic efficacy is frequently limited by the survival of residual tumor cells that can re‐enter the proliferative cycle. Our previous studies showed that residual cholangiocarcinoma cells can re‐enter the proliferative cycle following PDT accompanied by hypoxia‐induced activation of the HIF‐1α survival pathway and intracellular antioxidant programs. To address this residual viability, we developed a glutathione‐responsive targeted nanosystem (TSH NPs), in which the aggregation‐induced emission (AIE) photosensitizer TPA‐Ph‐RDN is conjugated to the chemotherapeutic agent hydroxycamptothecin (HCPT) via a disulfide linkage. This design enables HCPT to exert direct cytotoxic effects while simultaneously suppressing the HIF‐1α‐mediated hypoxic adaptation pathway, thereby reducing residual tumor viability and enhancing PDT efficacy. This strategy achieves a dual‐mechanism, two‐pronged therapeutic effect. Both in vitro and in vivo studies demonstrated that TSH NPs elicited markedly synergistic antitumor activity, highlighting their potential as a promising therapeutic approach for improving clinical outcomes in patients with eCCA.

## Graphical abstract

A GSH‐responsive AIE nanoplatform (TSH NPs) with RGD‐mediated tumor targeting co‐delivers a photosensitizer (TPA) and HCPT for extrahepatic cholangiocarcinoma. The system synergistically enhances PDT and chemotherapy by suppressing HIF‐1α/VEGF‐driven hypoxic adaptation, effectively overcoming PDT resistance. This multimodal strategy achieves potent antitumor efficacy, mitigates recurrence, and reduces systemic toxicity, offering a promising preclinical candidate.

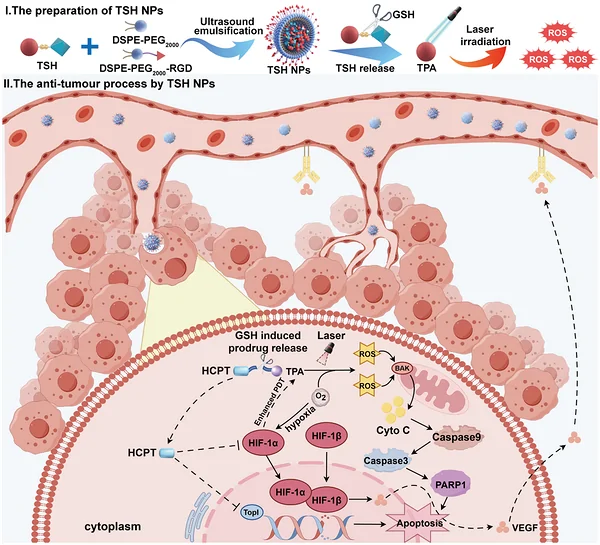

## 1 Introduction

Extrahepatic cholangiocarcinoma (eCCA) is an aggressive malignancy of the biliary tract with a poor prognosis and is typically diagnosed at an advanced stage because of nonspecific early symptoms and complex anatomical features, thereby precluding curative resection in most patients [1, 2, 3]. For these individuals, the combination of gemcitabine and cisplatin remains the first‐line treatment regimen; however, its clinical efficacy is limited by intrinsic drug resistance and systemic toxicity [4]. In addition, immunotherapy and targeted therapies are applicable only to a small subset of patients with specific molecular alterations, further restricting their overall clinical benefit [5, 6]. Photodynamic therapy (PDT) is a promising anticancer modality that has been recommended by the National Comprehensive Cancer Network as a standard palliative treatment for cholangiocarcinoma [7]. It is valued for its minimally invasive nature, high selectivity, low incidence of adverse effects, and lack of cross‐resistance with conventional chemotherapeutic agents [8]. The fundamental mechanism of PDT involves the light‐induced activation of photosensitizers, which subsequently generate reactive oxygen species (ROS) through interactions with intracellular oxygen [9, 10]. The resulting oxidative stress induces damage to subcellular organelles and critical biomolecules, such as DNA and proteins, ultimately leading to a triad of antitumor effects: direct tumor cell death, disruption of tumor vasculature, and activation of host immune responses [11].

Nevertheless, conventional photosensitizers are limited by poor photostability and the aggregation‐caused quenching (ACQ) effect, whereby ROS‐generating capacity decreases rather than increases at high concentrations or in aggregated states [12]. This limitation significantly hampers the clinical translation of many otherwise promising photosensitizers and substantially reduces the overall efficacy of PDT. In contrast, aggregation‐induced emission (AIE) photosensitizers exhibit enhanced luminescence efficiency and ROS generation in the aggregated state, effectively reversing the ACQ phenomenon [13, 14, 15]. Consequently, AIE photosensitizers have emerged as a highly promising direction for future PDT applications, offering substantial potential to improve both the efficiency and therapeutic outcomes of PDT [16]. For instance, Wang et al. [17] developed a series of donor–π–acceptor structured AIE molecules (DTTPy, OMeDTTPy, and BuDTTPy) that combine AIE properties with high fluorescence quantum yields and robust ROS generation, enabling effective in situ PDT for breast cancer under near‐infrared II fluorescence guidance. This work highlights the potential of AIE photosensitizers to overcome ACQ, enhance tumor‐specific accumulation, and integrate diagnostic imaging with therapeutic intervention.

Despite its ability to reduce tumor burden as a local palliative treatment, the efficacy of PDT is frequently limited by the persistence of residual tumor cells [18]. As documented in our previous in vivo studies [19, 20], pronounced hemorrhagic tumor regrowth was observed at treatment sites one week after PDT. Consistently, our in vitro experiments demonstrated that a subset of tumor cells exposed to sublethal PDT doses regained proliferative capacity [21], suggesting that surviving cells can mount adaptive responses that allow continued proliferation. Furthermore, accumulating evidence indicates that residual cancer cells, whether exposed to sublethal ROS levels or surviving incomplete tumor vascular shutdown, can activate a network of adaptive intracellular survival pathways, including HIF‐1α, NRF2, AP‐1, and ATF4 [22, 23]. Activation of these pathways promotes antioxidant system reconstruction, enhances cellular stress tolerance, and ultimately contributes to acute adaptive survival and subsequent tumor regrowth.

In the present study, the term “PDT resistance” is used operationally to describe a phenomenon distinct from classical acquired drug resistance. It refers to the acute adaptive survival of tumor cells after a single sublethal PDT session, including transient attenuation of oxidative‐stress‐induced killing and the subsequent proliferative recovery that leads to tumor regrowth. Increasing evidence suggests that this adaptive response is closely associated with rapid oxygen depletion within the tumor microenvironment during PDT irradiation, which induces hypoxic stress and activates adaptive cellular survival mechanisms that undermine therapeutic efficacy [22, 24]. During this process, PDT‐induced oxygen depletion is accompanied by activation and nuclear translocation of hypoxia‐inducible factor‐1α (HIF‐1α), along with upregulation of downstream pro‐angiogenic mediators such as vascular endothelial growth factor (VEGF) [25, 26]. Activation of this signaling cascade facilitates tumor vascular regeneration, metabolic reprogramming, and the overexpression of anti‐apoptotic proteins, thereby forming a classical hypoxia‐adaptive feedback loop [27]. Collectively, these molecular events have been linked to attenuated PDT‐induced apoptosis and enhanced tumor cell survival, proliferation, invasion, and metastasis, and may contribute to the adaptive survival and tumor recurrence observed after PDT.

Previous studies by Zeng et al. and Liu et al. demonstrated that co‐administration of HIF‐1α inhibitors using phospholipid‐based delivery systems effectively suppresses HIF‐1α signaling, thereby enhancing the antitumor efficacy of PDT and delaying tumor recurrence [28, 29]. Building upon these findings, the present study aims to develop a combination therapeutic strategy that both addresses residual tumor viability by modulating hypoxia‐associated pathways and exerts potent cytotoxic effects against eCCA cells. Hydroxycamptothecin (HCPT), a potent camptothecin analogue, exerts antitumor activity by targeting nuclear topoisomerase I (Top I), thereby inhibiting DNA replication and transcription [30]. Its clinically approved derivative, irinotecan, has been adopted as a second‐line chemotherapeutic agent for the treatment of eCCA [31]. In addition, accumulating evidence indicates that HCPT suppresses HIF‐1α synthesis, leading to downregulation of the HIF‐1α/VEGF signaling cascade and attenuation of hypoxia‐adaptive responses [32, 33]. By attenuating HIF‐1α‐associated hypoxic adaptation, HCPT may reduce residual tumor viability and improve therapeutic efficacy.

**SCHEME 1:**
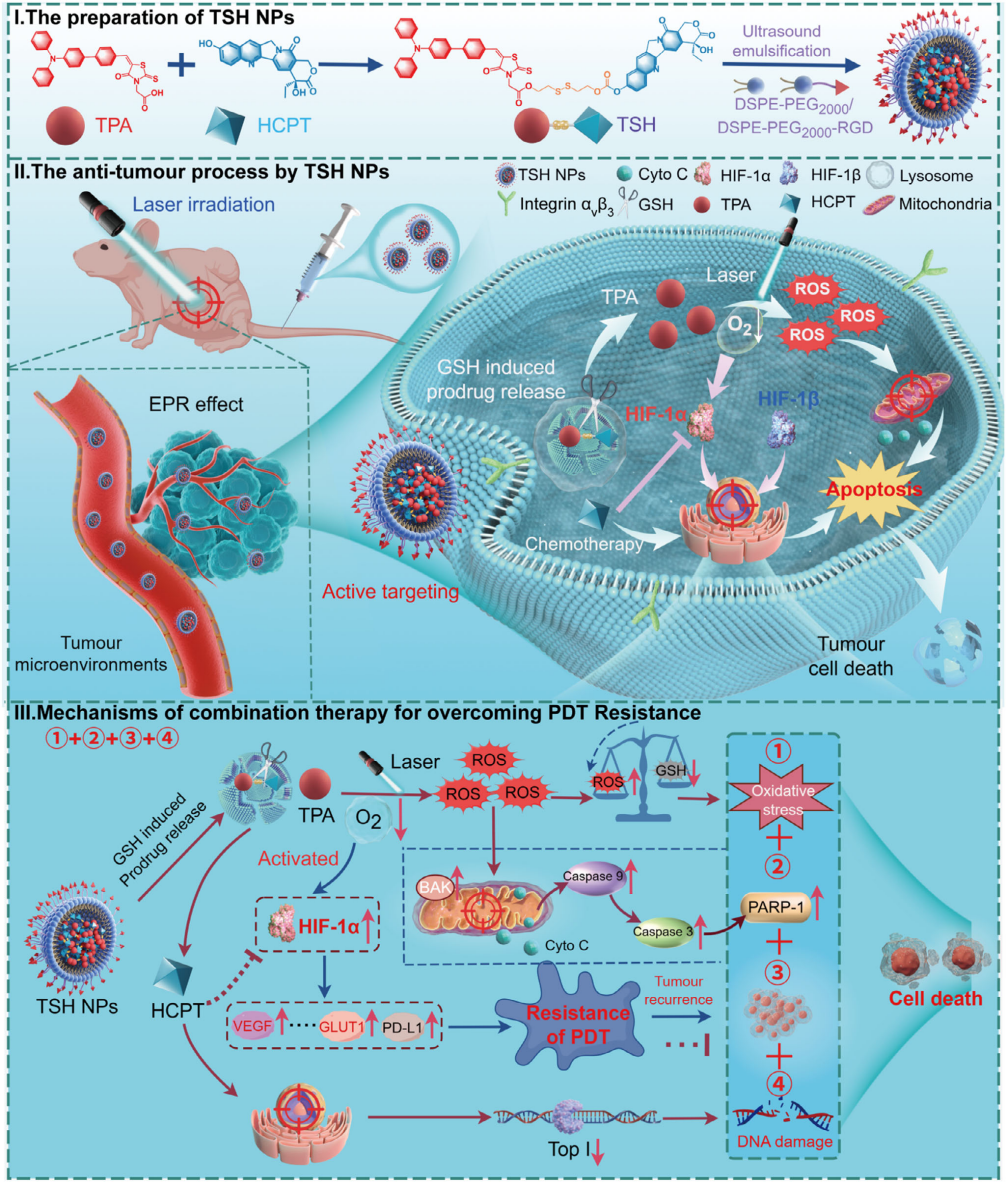
Illustration of the preparation and therapeutic mechanism of TSH NPs. (I) Synthetic route of TSH NPs. (II) Schematic representation of TSH NPs entering tumor cells following intravenous injection via the tail vein, enabling both photodynamic and chemotherapeutic effects. (III) Mechanistic depiction of the synergistic tumor‐killing effects mediated by PDT and HCPT.

Therefore, building upon our previous studies [19, 34], we developed a phospholipid–polyethylene glycol nanocarrier system employing DSPE‐PEG and DSPE‐PEG‐RGD as the amphiphilic outer shell. This system co‐encapsulates the AIE photosensitizer TPA‐Ph‐RDN (abbreviated as TPA) and the chemotherapeutic agent HCPT, an antagonist of the HIF‐1α/VEGF signaling pathway, via a disulfide linker to form a glutathione (GSH)‐responsive nanodrug designated as TPA‐SS‐HCPT nanoparticles (TSH NPs). This nanosystem targets integrin αvβ3 receptors that are overexpressed on eCCA cells and is expected to achieve efficient tumor accumulation through a combination of enhanced permeability and retention (EPR) effects and ligand–receptor‐mediated endocytosis. Within the intracellular microenvironment characterized by elevated GSH levels [35], the disulfide bonds undergo redox cleavage, consuming GSH and releasing TPA. Upon light irradiation, TPA generates ROS to induce photodynamic cytotoxicity. Concurrently, HCPT released from the nanosystem enters the nucleus, inhibits Top I, and suppresses DNA replication. Importantly, HCPT also attenuates PDT‐induced activation of the HIF‐1α pathway, thereby mitigating PDT resistance and enhancing overall therapeutic efficacy through synergistic PDT–chemotherapy effects (Scheme 1). Collectively, this study highlights the therapeutic potential of a combination strategy aimed at addressing residual tumor viability after PDT and represents the first investigation of the synergistic antitumor mechanism mediated by AIE‐photosensitizer‐based PDT combined with HCPT in eCCA cells, providing a solid foundation for the development of efficient and clinically translatable therapies for patients with eCCA.

## 2 Results and Discussion

### 2.1 Fabrication and Characteristics of TSH NPs

**FIGURE 1:**
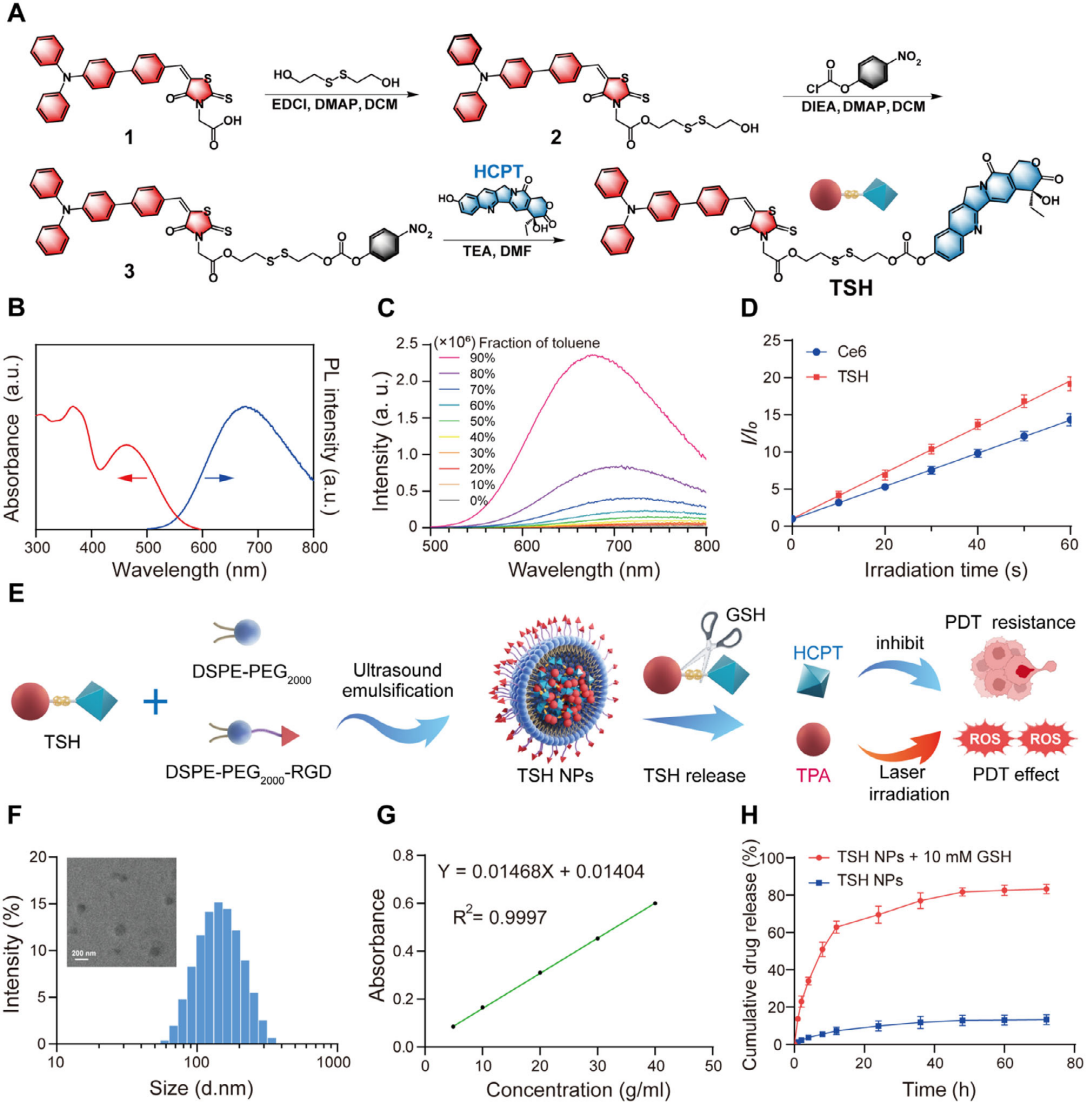
Fabrication and characteristics of TSH NPs. (A) Synthetic route of TSH. (B) UV–vis absorption spectrum and emission spectrum (*λ*_ex = 680 nm) of TSH. (C) Fluorescence spectra of TSH (10 µM) in DMSO/toluene mixtures with varying toluene fractions. (D) Evaluation of ROS generation based on the DCFH oxidation to DCF ratio (*I*/*I*_0_) under light irradiation (0.2 W/cm^2^), where *I*_0_ and *I* represent the fluorescence intensities of DCF (524 nm) before and after light exposure, respectively. (E) Preparation route and structural characteristics of TSH NPs. (F) Representative dynamic light scattering profile and transmission electron microscopy image of TSH NPs. (G) UV–vis absorption standard curves of TSH NPs. (H) Cumulative HCPT release from TSH NPs under physiological conditions in the presence or absence of GSH.

Based on the above strategy, we designed the targeted prodrug TSH by introducing disulfide and ester bonds to conjugate the AIE‐active photosensitizer TPA with the chemotherapeutic agent HCPT, thereby enabling GSH‐responsive drug release within tumor cells. As shown in Figure 1A, the synthesis of TSH was achieved through a three‐step process. First, compound 1 (TPA) was synthesized according to a previously reported method [36] and subsequently reacted with 1,2‐hydroxyethyl disulfide in the presence of EDCI and DMAP in anhydrous dichloromethane under a nitrogen atmosphere. After completion of the reaction, the mixture was subjected to extraction and silica gel column chromatography to obtain compound 2. Second, compound 2 was reacted with 4‐nitrophenyl chloroformate and DMAP, followed by the slow dropwise addition of DIEA at room temperature. The resulting product was purified by column chromatography to yield compound 3. Finally, compound 3 was coupled with HCPT in anhydrous DMF in the presence of TEA. The reaction mixture was then extracted, dried, and purified by column chromatography to afford the final product, TSH.

As shown in Figures S1–S4, the molecular structures of synthesized compounds 1 and 2, as well as the prodrug TSH, were confirmed by nuclear magnetic resonance (NMR) spectroscopy and high‐resolution mass spectrometry (HRMS). Subsequently, the absorption spectrum of TSH dissolved in DMSO was analyzed using a UV–vis spectrophotometer. As shown in Figure 1B, TSH exhibited broad absorption bands ranging from 300 to 600 nm, with two distinct absorption peaks at 370 and 460 nm, and a maximum emission wavelength at 680 nm. The absorption peak at 370 nm was primarily attributed to the HCPT moiety.

The AIE properties of TSH were subsequently evaluated. As shown in Figures S5 and 1C, the fluorescence intensity of TSH in pure DMSO was extremely weak. However, with increasing volume fractions of toluene, the photosensitizer molecules gradually aggregated, resulting in a pronounced enhancement of fluorescence intensity. Notably, when the toluene content reached 90%, the fluorescence intensity increased by approximately 74‐fold compared with that observed at 10% toluene. In addition, a gradual blue shift in the emission spectrum of TSH was observed with increasing toluene content. These results collectively confirmed that the prodrug TSH exhibits typical AIE characteristics.

As the generation of ROS is a critical parameter for evaluating the efficacy of photosensitizers in PDT, chlorin e6 (Ce6), a widely recognized and clinically used photosensitizer, was employed as a reference control. To quantitatively compare the ROS‐generating efficiencies of TSH and Ce6 under identical conditions, the commercially available fluorescent probe DCFH was used. As shown in Figures S6 and S7, upon irradiation with white light at the same power density, both 10 µM TSH and 10 µM Ce6 solutions exhibited increased DCFH fluorescence intensity, indicating ROS generation. Notably, the fluorescence signal induced by TSH was markedly stronger than that of Ce6 (Figure 1D), confirming the superior ROS‐generating capability of TSH.

Following the successful synthesis and validation of the photosensitizing properties of the prodrug TSH, DSPE‐PEG_2000_‐RGD and DSPE‐PEG_2000_ were employed as nanocarriers to encapsulate the prodrug TSH and the photosensitizer TPA, yielding TSH NPs and TPA nanoparticles (TPA NPs), respectively. During the self‐assembly process, the hydrophobic DSPE segments interacted with the hydrophobic prodrug TSH to form the nanoparticle core, while the hydrophilic PEG chains extended into the aqueous phase, providing steric stabilization. This nanoparticle formulation confined the photosensitizer–prodrug conjugate within a hydrophobic microenvironment, promoting tight molecular packing that enhanced nanoparticle stability and facilitated efficient ROS generation. The preparation process of TSH NPs is illustrated in Figure 1E.

The morphology and hydrodynamic diameter of the nanoparticles were characterized by transmission electron microscopy (TEM) and dynamic light scattering (DLS). As shown in Figures 1F and S8, both TSH NPs and TPA NPs exhibited a uniform spherical morphology with an average diameter of approximately 100 nm, which is favorable for EPR‐mediated passive accumulation in tumor tissues. In addition, the encapsulation efficiency (EE) and drug loading capacity (DLC) of TPA and TSH were evaluated using UV–vis spectrophotometry based on their respective absorption coefficients. As shown in Figures S9 and 1G, the EE and DLC of TPA NPs were 72.51% and 6.76%, respectively, whereas those of TSH NPs were 81.34% and 7.52%, respectively. As shown in Figure S10, the activation and release kinetics of HCPT from GSH‐responsive TSH were monitored by fluorescence spectroscopy. The fluorescence intensity at 587 nm increased progressively over time, which was attributed to the caging of the phenolic hydroxyl groups of HCPT within TSH, resulting in transient quenching of HCPT fluorescence. Upon exposure to GSH, the disulfide bonds were cleaved and the phenolic hydroxyl groups were restored, thereby reactivating the intramolecular charge transfer (ICT) process of HCPT and turning on its fluorescence signal. These results confirm that TSH undergoes GSH‐triggered cleavage to release HCPT. Subsequently, high‐performance liquid chromatography (HPLC) was employed to quantitatively analyze HCPT release from TSH NPs. As shown in Figure 1H, only a minimal amount of HCPT was released from TSH NPs after 72 h of incubation under plasma‐mimicking conditions (pH 7.4). In contrast, under simulated intracellular tumor conditions containing 10 mM GSH, HCPT release was markedly accelerated, with cumulative release approaching 80% within 48 h. These results demonstrate that HCPT can be efficiently released from TSH NPs in response to GSH, confirming the GSH‐responsive behavior and favorable release profile of the nanosystem. Finally, the ROS‐generating capability of TSH NPs was further evaluated. As shown in Figure S11, TSH NPs efficiently generated ROS upon light irradiation, irrespective of the presence or absence of GSH.

### 2.2 Cellular Internalization and Intracellular Localization of TSH NPs

**FIGURE 2:**
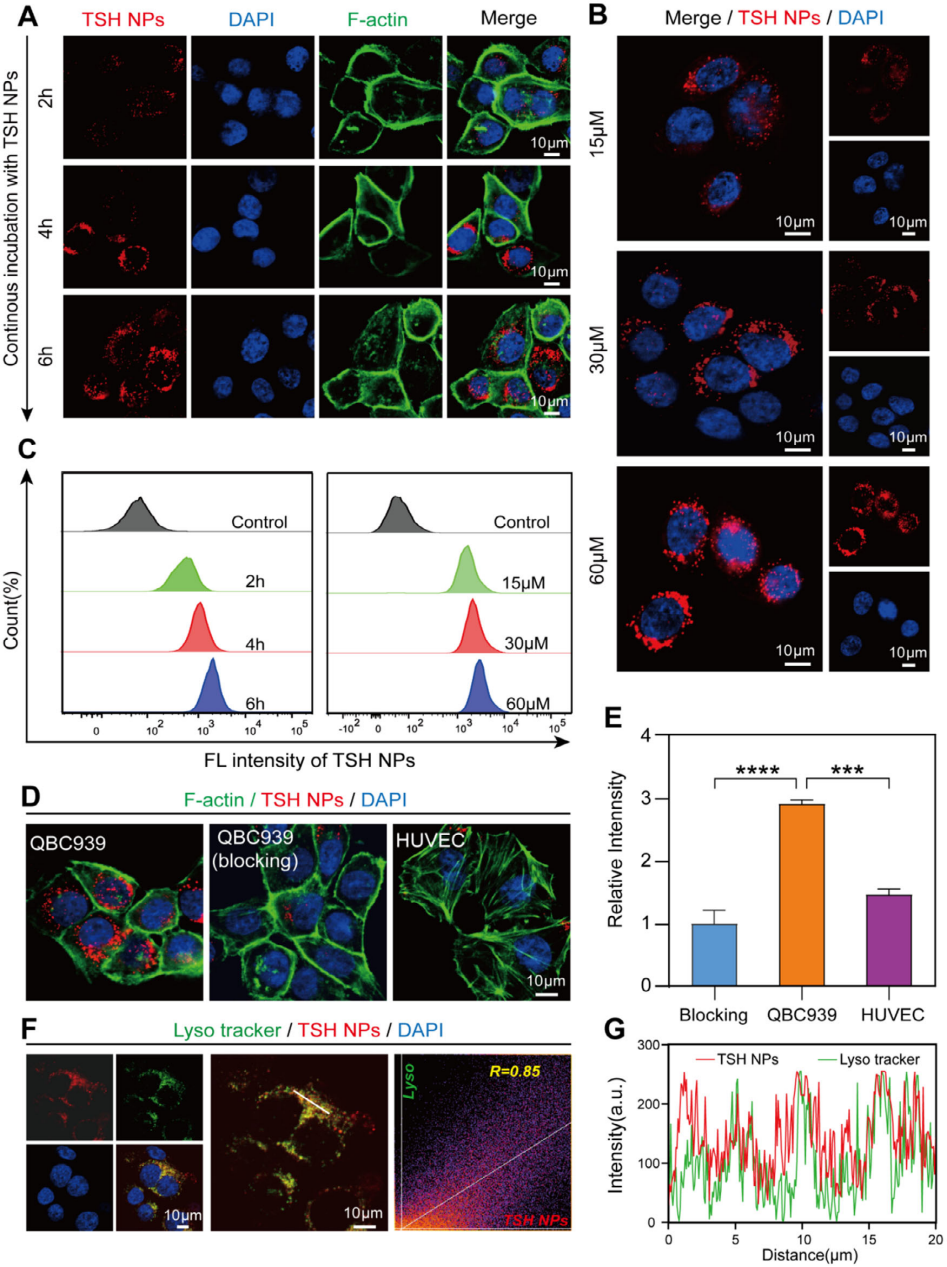
Investigation of the uptake mechanism and subcellular distribution of TSH NPs. (A) Representative CLSM images of QBC939 cells after incubation with TSH NPs for different durations ([TSH NPs] = 30 µM). (B) CLSM images of QBC939 cells after 6 h of incubation with TSH NPs at different concentrations. (C) Quantitative flow cytometric analysis of intracellular fluorescence intensity demonstrating time‐ and concentration‐dependent uptake of TSH NPs in QBC939 cells. (D) CLSM images showing TSH NP internalization after 6 h of incubation in QBC939 cells, cilengitide‐pretreated QBC939 cells, and HUVECs. (E) Quantitative fluorescence analysis comparing uptake efficiency among QBC939 cells, cilengitide‐pretreated QBC939 cells, and HUVECs. (F) Fluorescence co‐localization images and Pearson correlation analysis illustrating the intracellular distribution of TSH NPs relative to lysosomes in QBC939 cells. (G) Fluorescence co‐localization analysis of TSH NPs with cellular lysosomes.

Following confirmation of the stability and GSH‐triggered drug release properties of TSH NPs, their intracellular uptake was investigated in QBC939 cells, which is a prerequisite for effective therapeutic activity. Confocal laser scanning microscopy (CLSM) was employed to visualize and monitor the time‐dependent internalization of TSH NPs in QBC939 cells. As shown in Figure 2A,B, TSH NPs began to internalize into cells at approximately 2 h, and intracellular fluorescence intensity increased progressively with both incubation time and nanoparticle concentration. Pronounced accumulation of red fluorescence was observed after 6 h of incubation. As shown in Figure 2C, quantitative analysis by flow cytometry (FCM) further corroborated the CLSM observations, demonstrating that TSH NPs were internalized by QBC939 cells in a time‐ and concentration‐dependent manner. This efficient cellular uptake was presumed to occur primarily via receptor‐mediated endocytosis, attributed to specific interactions between ligands on the surface‐functionalized TSH NPs and corresponding receptors expressed on the tumor cell membrane.

Our previous study demonstrated that integrin αvβ3 is overexpressed in QBC939 cells and tumor tissues, whereas its expression is minimal in most normal organs [19]. Accordingly, RGD peptide‐functionalized TSH NPs were hypothesized to facilitate active targeting and receptor‐mediated endocytosis in tumor cells, thereby enhancing the specificity and efficiency of intracellular drug delivery. To validate this hypothesis, QBC939 cells were incubated with TSH NPs under three conditions: without pretreatment, with cilengitide pretreatment (a specific integrin αvβ3 inhibitor), and in comparison with human umbilical vein endothelial cells (HUVECs) as a non‐tumor control. As shown in Figure 2D, CLSM images obtained under identical excitation conditions revealed a marked reduction in red fluorescence intensity in both cilengitide‐pretreated QBC939 cells and HUVECs, indicating significantly decreased cellular uptake of TSH NPs. Quantitative analysis using ImageJ confirmed that the intracellular fluorescence intensity in untreated QBC939 cells was approximately 2.9‐fold and 2.5‐fold higher than that observed in the cilengitide‐treated and HUVEC groups, respectively (Figure 2E). Collectively, these results provide strong evidence for the enhanced targeting specificity of TSH NPs toward QBC939 cells.

To further elucidate the intracellular trafficking pathway of TSH NPs following internalization, QBC939 cells were incubated with TSH NPs and subsequently co‐stained with LysoTracker Green, a fluorescent probe specific for acidic organelles such as endosomes and lysosomes. As shown in Figure 2F,G, the red fluorescence signal of TSH NPs exhibited strong co‐localization with lysosomal fluorescence. Quantitative co‐localization analysis yielded a Pearson correlation coefficient of 0.85, indicating that TSH NPs are predominantly trafficked through the endosome–lysosome pathway.

### 2.3 Synergistic Cytotoxic Effects of TSH NPs In Vitro

**FIGURE 3:**
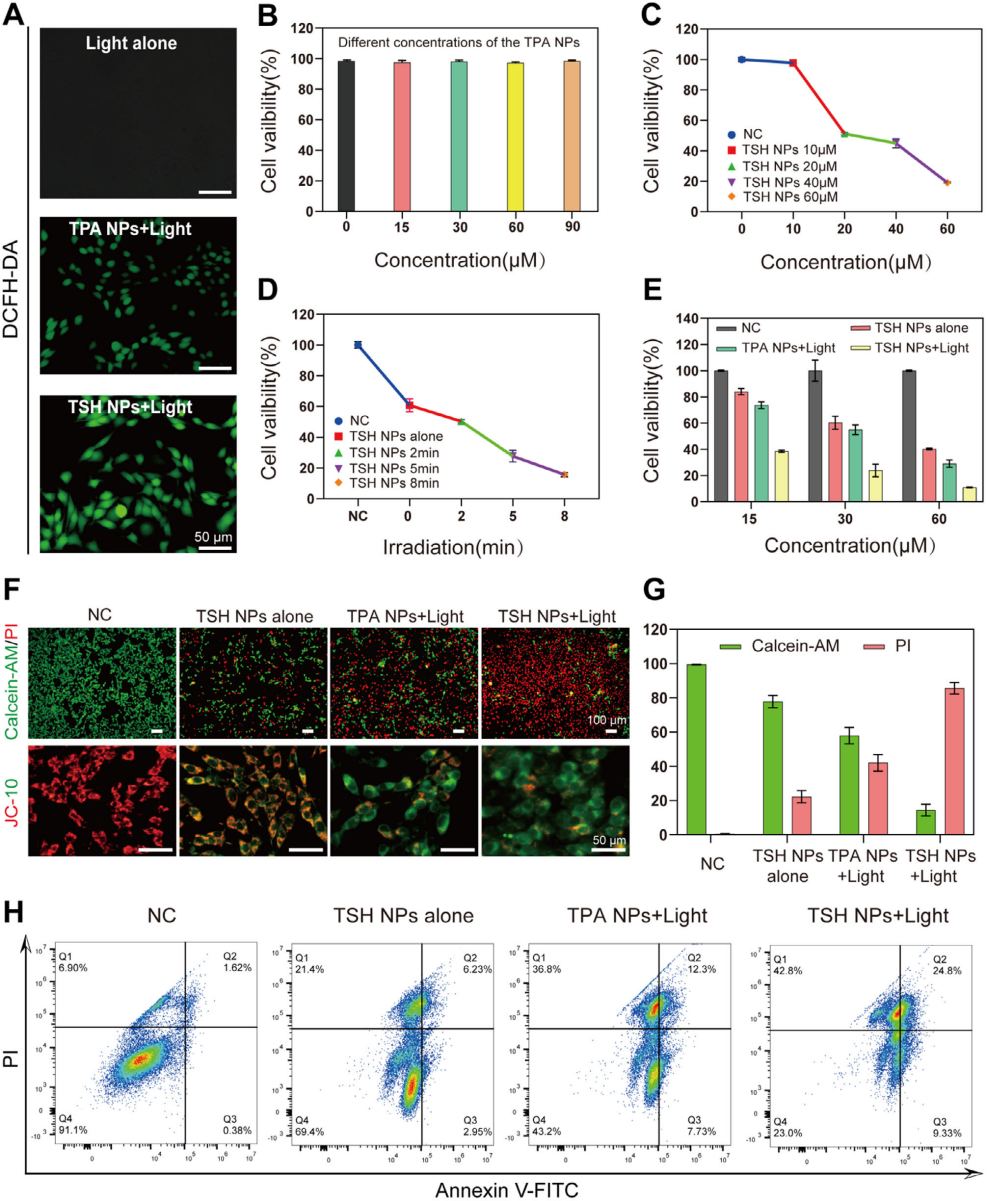
Synergistic cytotoxic effects of TSH NPs in vitro. (A) Intracellular ROS generation in QBC939 cells following treatment with light alone, TPA NPs plus light, and TSH NPs plus light. (B) Dark cytotoxicity of TPA NPs at different concentrations. (C) Cell viability of QBC939 cells after incubation with varying concentrations of TSH NPs under dark conditions. (D) Cell viability of QBC939 cells treated with TSH NPs under different durations of light irradiation. (E) Comparative cytotoxic effects against QBC939 cells among the negative control group, chemotherapy‐only group, TPA NPs plus light group, and TSH NPs plus light group. (F) Calcein‐AM/PI live/dead cell staining (top) and JC‐10 mitochondrial membrane potential analysis (bottom) in QBC939 cells following treatments in the negative control group, chemotherapy‐only group, TPA NPs plus light group, and TSH NPs plus light group. (G) Quantitative analysis of live/dead staining in QBC939 cells across different treatment groups. (H) Apoptosis rates of QBC939 cells in the negative control group, chemotherapy‐only group, TPA NPs plus light group, and TSH NPs plus light group, as determined by Annexin V‐FITC/PI staining.

The above experiments demonstrated that TSH NPs were efficiently internalized by QBC939 cells via receptor–ligand‐mediated active targeting, followed by endosomal–lysosomal trafficking. Notably, optimal cellular uptake was observed after 6 h of incubation. Based on these findings, we further investigated whether TSH NPs could enable a combined PDT and chemotherapy strategy in vitro to enhance therapeutic efficacy. ROS are regarded as key indicators of cellular structural damage and impaired integrity, and the intracellular ROS levels generated upon light irradiation directly determine the therapeutic effectiveness of PDT. As shown in Figures 3A, S12, and S13, intracellular ROS production in the different treatment groups was evaluated using the fluorescent probe 2′,7′‐dichlorodihydrofluorescein diacetate (DCFH‐DA). Both the PDT monotherapy group (TPA NPs + light) and the combination therapy group (TSH NPs + light) exhibited robust ROS generation, whereas negligible ROS levels were detected in the chemotherapy‐only group (TSH NPs without irradiation) and the light‐only group. Importantly, the combination treatment group showed significantly higher ROS generation than the PDT monotherapy group. This enhancement may be attributed to both the intrinsic ROS‑generating capacity of the AIE photosensitizer and the GSH depletion caused by disulfide bond reduction within TSH NPs, with the latter impairing cellular antioxidant defenses and thereby exacerbating oxidative stress.

Subsequently, the in vitro antitumor efficacy of each treatment group was quantitatively assessed using the Cell Counting Kit‐8 (CCK‐8) assay. As shown in Figure 3B, no significant cytotoxicity was observed in QBC939 cells after 24 h of incubation with varying concentrations (0–90 µM) of TPA NPs under dark conditions, indicating favorable cytocompatibility and providing a suitable safety margin for subsequent photodynamic applications. In contrast, as shown in Figure 3C, TSH NPs exhibited marked antitumor activity against QBC939 cells under dark conditions. This cytotoxic effect was concentration‐dependent, with an estimated IC_50_ of approximately 30 µM, suggesting that intracellular release of the chemotherapeutic agent HCPT effectively inhibited DNA replication following nanoparticle internalization.

To further evaluate the therapeutic efficacy of TSH NPs under light irradiation, their cytotoxic effects were assessed and compared with those of TPA NPs at equivalent concentrations. As shown in Figure 3D,E, cell viability in the TSH NP‐treated group decreased progressively with increasing light irradiation time, indicating a time‐dependent phototoxic response. Notably, under identical conditions, the TSH NPs + light exhibited significantly greater antitumor efficacy than either the TPA NPs + light or the chemotherapy‐only group (TSH NPs without light). According to the Chou–Talalay method, the combination index (CI) for TSH NPs + light at the 50% inhibition level was calculated to be 0.622, indicating a synergistic effect between PDT and HCPT chemotherapy in QBC939 cells. To visually evaluate cytotoxic outcomes across different treatment groups, calcein‐AM/propidium iodide (PI) dual staining was performed to distinguish live and dead cells (Figure 3F,G). These results clearly demonstrated that the combination therapy induced substantial cell death, highlighting a pronounced synergistic cytotoxic effect.

Encouraged by these findings, we further explored the potential mechanisms underlying the synergistic induction of apoptosis by the combination treatment. Previous studies have established that mitochondria play a central role in apoptosis, and that photosensitizers targeting lysosomes can initiate endogenous apoptosis via mitochondria‐mediated pathways [20, 34]. Early events in this process include dissipation of the mitochondrial membrane potential (Δ*Ψ*m), increased membrane permeability, and subsequent release of pro‐apoptotic factors [37]. Given the lysosome‐targeting characteristics of TSH NPs, alterations in mitochondrial membrane potential were assessed shortly after light irradiation using the JC‐10 fluorescent probe. As shown in Figure 3F, the most pronounced mitochondrial depolarization occurred in the combination treatment group, reflecting severe mitochondrial dysfunction. In contrast, only a mild decrease in Δ*Ψ*m was observed in the chemotherapy‐only group, indicating a limited apoptotic response.

Finally, total cell death (including apoptosis and necrosis) was quantitatively analyzed using Annexin V‐FITC/PI staining 24 h after treatment (Figure 3H). The combination therapy group exhibited the highest rate of total cell death, followed by the PDT monotherapy and chemotherapy‐only groups, all of which showed significantly higher levels than the negative control. Collectively, these results indicate that the combination of PDT and the chemotherapeutic agent HCPT synergistically enhances overall cell death, thereby substantially amplifying the overall antitumor efficacy of TSH NPs.

### 2.4 HCPT Attenuates PDT‐Induced Adaptive Survival via HIF‐1α‐Associated Pathways

**FIGURE 4:**
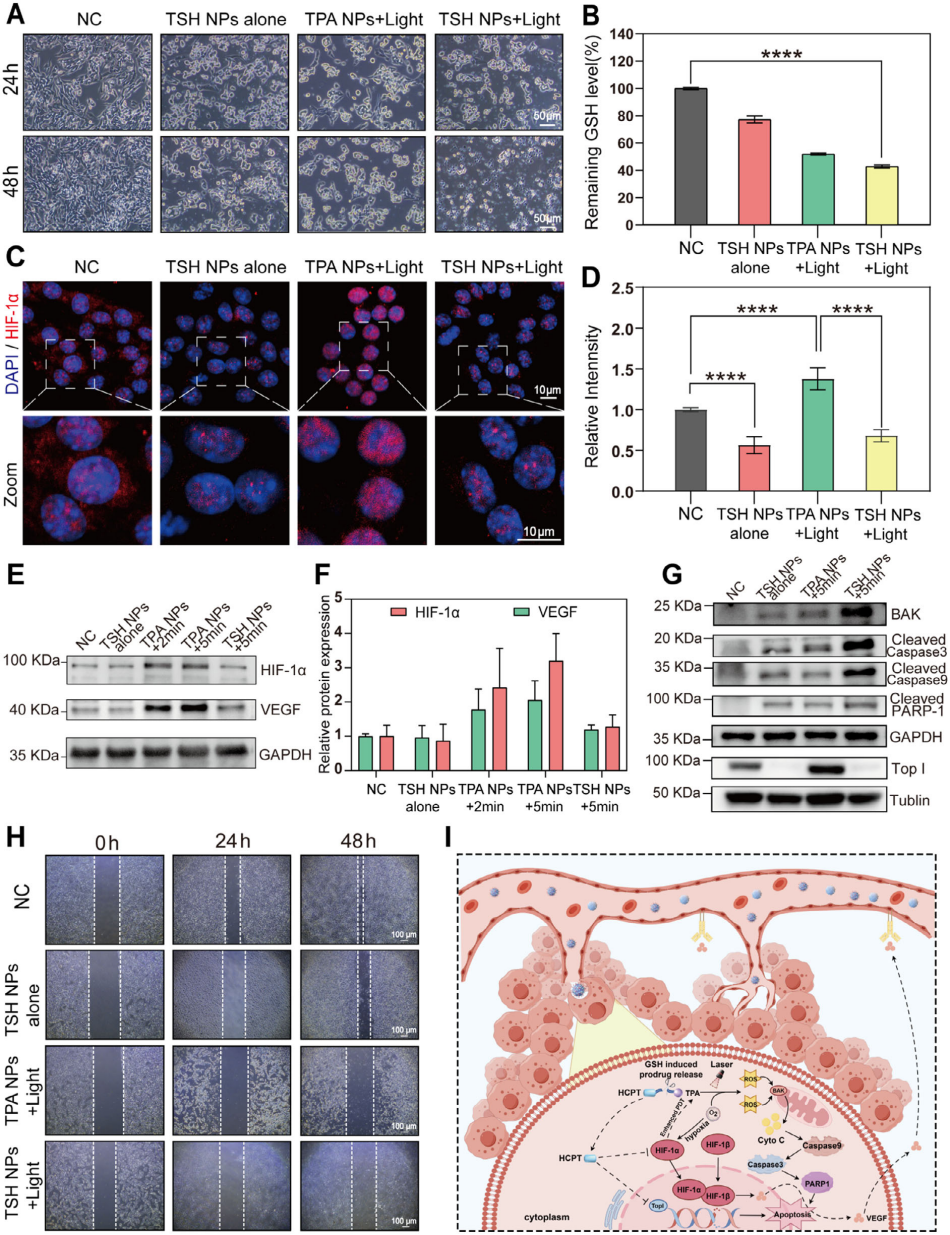
HCPT attenuates PDT‐induced adaptive survival via HIF‐1α‐associated pathways. (A) Representative time‐course images illustrating morphological changes in QBC939 cells following different treatments: negative control, chemotherapy alone, TPA NPs + laser irradiation, and TSH NPs + laser irradiation at 24 h and 48 h. (B) Quantification of intracellular GSH levels after treatment in the four groups. (C) Immunofluorescence images showing HIF‐1α expression in QBC939 cells under different treatment conditions. (D) Quantitative analysis of HIF‐1α immunofluorescence intensity. (E) Western blot analysis of HIF‐1α and VEGF expression following the indicated treatments. (F) Quantitative analysis of HIF‐1α and VEGF protein levels. (G) Western blot analysis of apoptosis‐related and nuclear damage–associated proteins (BAK, caspase‐3, caspase‐9, PARP‐1, Top I) with GAPDH and tubulin as loading controls. (H) Representative images from the wound‐healing assay illustrating cell migration under different treatments. (I) Schematic diagram of the proposed synergistic therapeutic mechanism of TSH NPs.

The above results demonstrated that combination treatment mediated by TSH NPs induced significantly greater cytotoxicity, oxidative stress, and pro‐apoptotic effects than PDT monotherapy. These findings suggest that this combinatorial strategy holds considerable potential for improving antitumor efficacy. To further assess the durability of the cytotoxic effects, a time‐dependent morphological analysis of QBC939 cells was conducted across the four experimental groups following treatment. As shown in Figures 4A and S14, extensive cell death was observed in the combination treatment group upon light irradiation. In contrast, cells treated with PDT monotherapy or chemotherapy alone displayed only partial apoptotic features, such as membrane shrinkage, at 24 and 48 h post‐treatment. Notably, a subset of these cells exhibited signs of morphological recovery and resumed proliferation. This phenomenon was consistent with our previous observations [21], in which residual tumor cells demonstrated a tendency toward relapse or reactivation following PDT monotherapy. These findings prompted further investigation into the mechanisms underlying this adaptive survival and the potential synergistic role of HCPT in reducing residual tumor viability. This adaptive survival involves the activation of multiple stress‐response pathways in tumor cells following initial PDT‐induced damage, and has been associated with increased tolerance to oxidative stress and modulation of the tumor microenvironment. This phenomenon has been associated with reduced therapeutic efficacy, tumor recurrence, and treatment failure [38]. Both the published literature and our previous studies have suggested that this adaptive response is closely associated with enhanced oxidative stress tolerance mechanisms and the aggravation of local hypoxia caused by sustained oxygen consumption during PDT [39, 40]. Accordingly, to explore the regulatory mechanisms underlying the enhanced therapeutic effects, we first quantified intracellular GSH levels across the four experimental groups to evaluate the status of the antioxidant system. As shown in Figure 4B, GSH levels were markedly depleted in the PDT group after photoactivation, owing to excessive ROS generation. In the chemotherapy group, a modest reduction in GSH was observed, likely resulting from the reaction between intracellular GSH and the disulfide bonds in TSH NPs. Notably, the combination treatment group exhibited the most pronounced GSH depletion, indicating a strong synergistic effect between PDT and HCPT in elevating intracellular oxidative stress.

Furthermore, protein expression levels of HIF‐1α and its downstream pro‐angiogenic factor VEGF were evaluated by Western blot analysis. As shown in Figure 4E,F, the expression of both HIF‐1α and VEGF in the PDT monotherapy group increased in a time‐dependent manner following irradiation. By contrast, both the chemotherapy group and the combination treatment group exhibited significant downregulation of these proteins, consistent with the immunofluorescence results. These findings indicate that PDT monotherapy induces a hypoxic microenvironment and activates the HIF‐1α–mediated signaling cascade, thereby facilitating tumor cell escape from PDT‐induced cytotoxicity. Importantly, the introduction of HCPT counteracted this effect by suppressing HIF‐1α expression, along with attenuation of hypoxia‐associated signaling and reduced residual tumor viability.

To further elucidate the mechanisms underlying cell death induced by the combination therapy, we examined the expression of proteins associated with the mitochondrial apoptotic pathway. As shown in Figures 4G and S15–S19, the combination treatment group exhibited significantly elevated expression levels of pro‐apoptotic proteins, including BAK, caspase‐3, caspase‐9, and the downstream effector PARP‐1, compared with both the chemotherapy‐alone and PDT monotherapy groups. Moreover, HCPT‐containing treatments effectively suppressed Top I expression, indicating enhanced DNA damage and impaired DNA repair capacity, which may further promote apoptosis.

To observe the effects of combination therapy on tumor cell repopulation in a denuded area, a wound‐healing (scratch) assay was performed. As shown in Figure 4H, untreated QBC939 cells exhibited rapid migration and effectively repopulated the scratched area. In contrast, all treatment groups showed reduced repopulation, with the combination treatment group exhibiting the most pronounced reduction (approximately 90% inhibition of wound closure). Given the concomitant cytotoxicity and apoptosis, however, this observation should be interpreted as a composite effect of cell death and potential migration impairment rather than as a pure anti‐migratory effect.

In summary, the combination therapy of PDT and HCPT mediated by TSH NPs exerts synergistic antitumor effects through a dual mechanism. On one hand, PDT‐induced ROS cause oxidative damage and trigger apoptosis; on the other hand, HCPT targets topoisomerase I to inhibit DNA repair while simultaneously attenuating hypoxia‐associated signaling pathways at the molecular level, thereby reducing residual tumor viability after PDT. By concurrently acting on two critical organelles, namely the mitochondria and the nucleus, this combinatorial strategy not only markedly enhances antitumor efficacy but also suppresses tumor recurrence, angiogenesis, and metastatic progression, as illustrated in Figure 4I.

### 2.5 HIF‐1α‐Associated Transcriptional Reprogramming Contributes to Adaptive Survival After PDT and Its Attenuation by TSH NPs

**FIGURE 5:**
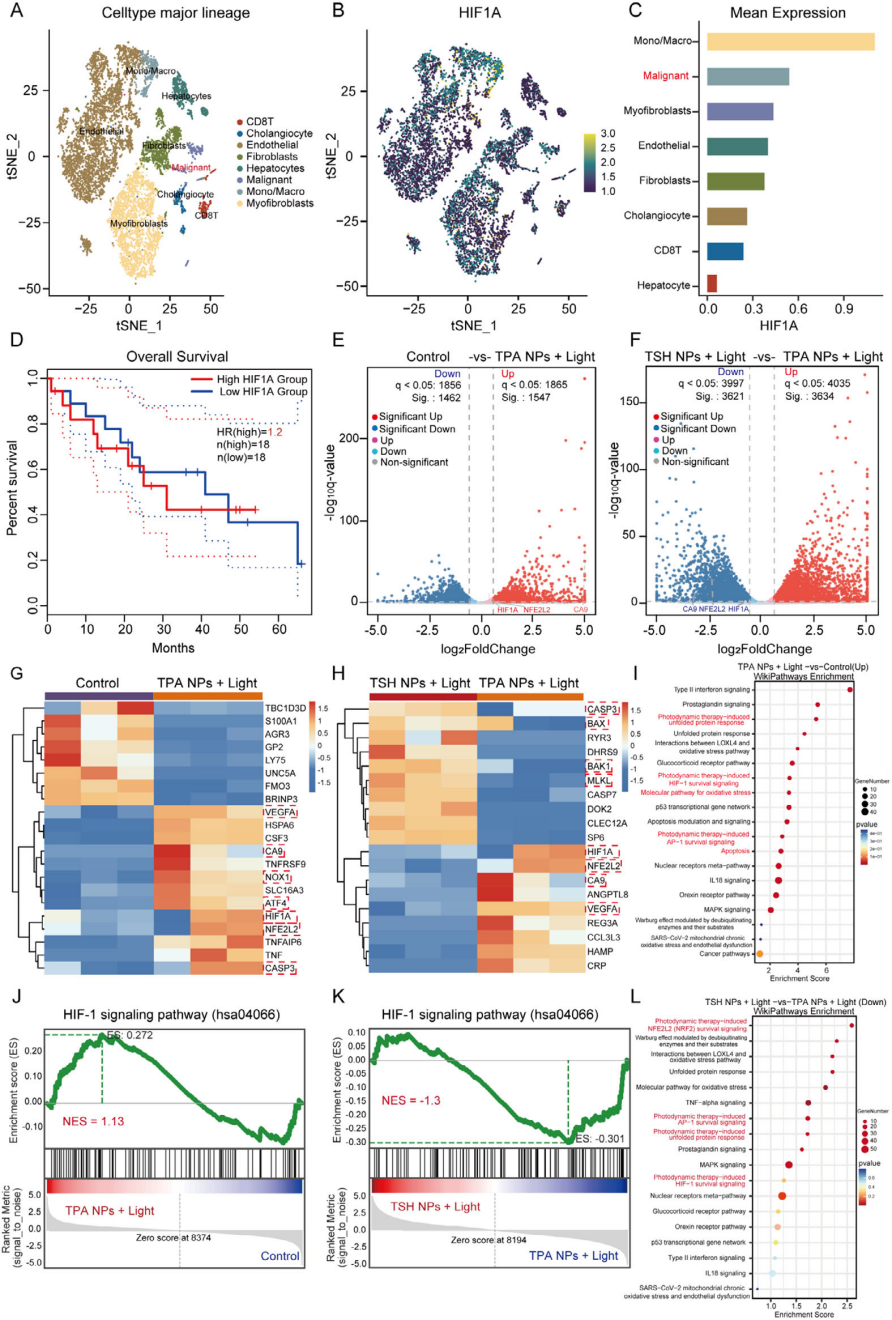
HIF‐1α‐associated transcriptional reprogramming contributes to adaptive survival after PDT and its attenuation by TSH NPs. (A) t‐SNE plot of single‐cell clustering in bile duct cancer, with distinct colors representing different cell types. (B) t‐SNE plot showing the expression distribution of HIF‐1A across cell populations. (C) Bar chart depicting HIF‐1A expression abundance in different cell types. (D) Kaplan–Meier survival analysis of HIF‐1A expression in TCGA data, with groups compared using the log‐rank test. HR (High exp) indicates the hazard ratio of the high‐expression group relative to the low‐expression group. (E) Volcano plot showing differentially expressed genes in QBC939 cells treated with TPA NPs + light compared with the control group. (F) Volcano plot showing differential gene expression between the TSH NPs + light group and the TPA NPs + light group. (G) Heatmap illustrating induction of hypoxia‐response, antioxidant, and anti‐apoptotic genes following PDT monotherapy. (H) Heatmap showing suppression of hypoxia‐adaptive genes and upregulation of apoptosis‐related effectors in the TSH NPs combination group. (I) WikiPathways enrichment analysis demonstrating activation of PDT‐induced survival programmes following TPA NPs + light treatment. (J) GSEA plot showing positive enrichment of the HIF‐1 signaling pathway in the PDT monotherapy group. (K) GSEA showing negative enrichment of the HIF‐1 signaling pathway following TSH NPs treatment. (L) WikiPathways enrichment analysis of downregulated genes in the TSH NPs + light group relative to the TPA NPs + light group.

The above experiments provide protein‐level evidence demonstrating that HIF‐1α is associated with adaptive survival after PDT. Therefore, to further elucidate the role of HIF‐1α in adaptive survival after PDT and to clarify how TSH NPs attenuate this phenotype at the transcriptional level, we performed an integrated analysis incorporating single‐cell profiling, whole‐transcriptome sequencing, and clinical prognostic evaluation. This approach was designed to comprehensively characterize the HIF‐1α‐associated adaptive survival network and to assess its therapeutic tractability. To this end, single‐cell transcriptomic analysis was first conducted to characterize the cell type–specific expression landscape of HIF‐1A in eCCA tissues, as shown in Figure 5A–C.

Among the major cholangiocyte‐associated cellular compartments, HIF‐1A expression was enriched in malignant epithelial cells compared with hepatocytes, fibroblasts, immune cells, and other stromal populations (Figure 5B,C). Consistently, analysis of publicly available transcriptomic datasets revealed a significant upregulation of HIF‐1A in tumor tissues relative to matched adjacent non‐tumorous samples (Figure S20), underscoring its fundamental involvement in tumor‐specific stress adaptation and metabolic reprogramming. Moreover, survival analysis demonstrated that patients with high HIF‐1A expression exhibited significantly shorter overall survival than those with low expression (Figure 5D). Collectively, these findings indicate that HIF‐1A is not only a central determinant of eCCA cellular pathophysiology but also a critical molecular driver associated with tumor progression and unfavorable clinical outcomes.

Subsequently, to examine the transcriptional changes associated with adaptive survival after PDT, we conducted integrated whole‐transcriptome profiling combined with pathway enrichment analysis. Differences in gene expression across the three sample groups, together with sample correlation and similarity analyses, are presented in Supplementary Figure S21–S24. As shown in Figure 5E, TPA NPs + light induced broad transcriptional activation of genes associated with hypoxic adaptation, antioxidant defense, and anti‐apoptotic signaling compared with the control group. Notably, key regulatory molecules, including HIF1A, NFE2L2 (NRF2), ATF4, CA9, and VEGFA, were markedly upregulated (Figure 5G). This transcriptional profile is consistent with the possibility that PDT‑induced oxygen depletion and ROS generation may elicit an adaptive response that could contribute to cellular protection under therapeutic stress. Such enrichment patterns have been previously linked to tumor cell persistence after PDT, although this interpretation from transcriptomic data alone remains correlative.

WikiPathways and Gene Ontology enrichment analyses further demonstrated that PDT monotherapy significantly activated multiple survival‐related pathways, including the PDT‐induced unfolded protein response, AP‐1 survival signaling, HIF‐1 survival signaling, the NRF2‐mediated oxidative stress tolerance pathway, and several programmes involved in anti‐apoptosis and metabolic homeostasis (Figures 5I and S25). Gene set enrichment analysis further corroborated these findings, revealing positive enrichment of the HIF‐1 signaling pathway in the PDT monotherapy group (NES = 1.13; Figure 5J). Collectively, these transcriptomic findings raise the hypothesis that HIF‑1α‑associated hypoxic adaptation may play a role in adaptive survival after PDT, with the corresponding protein–protein interaction network shown in Figure S26.

In contrast, as shown in Figure 5F, transcriptional profiling of tumors treated with the TSH NPs + light revealed a markedly distinct molecular landscape compared with PDT monotherapy. Key regulators of hypoxic adaptation and antioxidant defense, including HIF1A, VEGF, CA9, and NFE2L2, were substantially downregulated, whereas essential pro‐apoptotic mediators such as CASP3, CASP7, BAK1, and MLKL were robustly upregulated (Figure 5H). Consistently, pathway enrichment analyses showed that the combination treatment suppressed multiple survival and stress‐tolerance programmes, including PDT‐induced HIF‐1, NRF2, and AP‐1 signaling, as well as several oxidative stress‐relief pathways (Figures 5L and S27). Concurrently, signaling programmes associated with the p53 regulatory axis, TNF‐α signaling, programmed necrosis, and inflammatory responses were activated.

This coordinated shift across multiple signaling pathways indicates that the combination treatment reorients cellular fate from adaptive stress responses toward an irreversible apoptotic trajectory, with the corresponding protein–protein interaction network shown in Figure S28. Gene set enrichment analysis further supported these observations, demonstrating significant negative enrichment of the HIF‐1 signaling pathway following combination treatment (NES = −1.3; Figure 5K). Together, our transcriptomic data are consistent with our earlier protein and immunofluorescence findings, suggesting that HCPT may attenuate PDT‑induced hypoxic compensation, potentially through suppression of HIF‑1α‑related transcriptional programmes and attenuation of VEGF‑dependent signaling.

### 2.6 In Vivo Biosafety and Tumor‐Targeting Capability of TSH NPs

**FIGURE 6:**
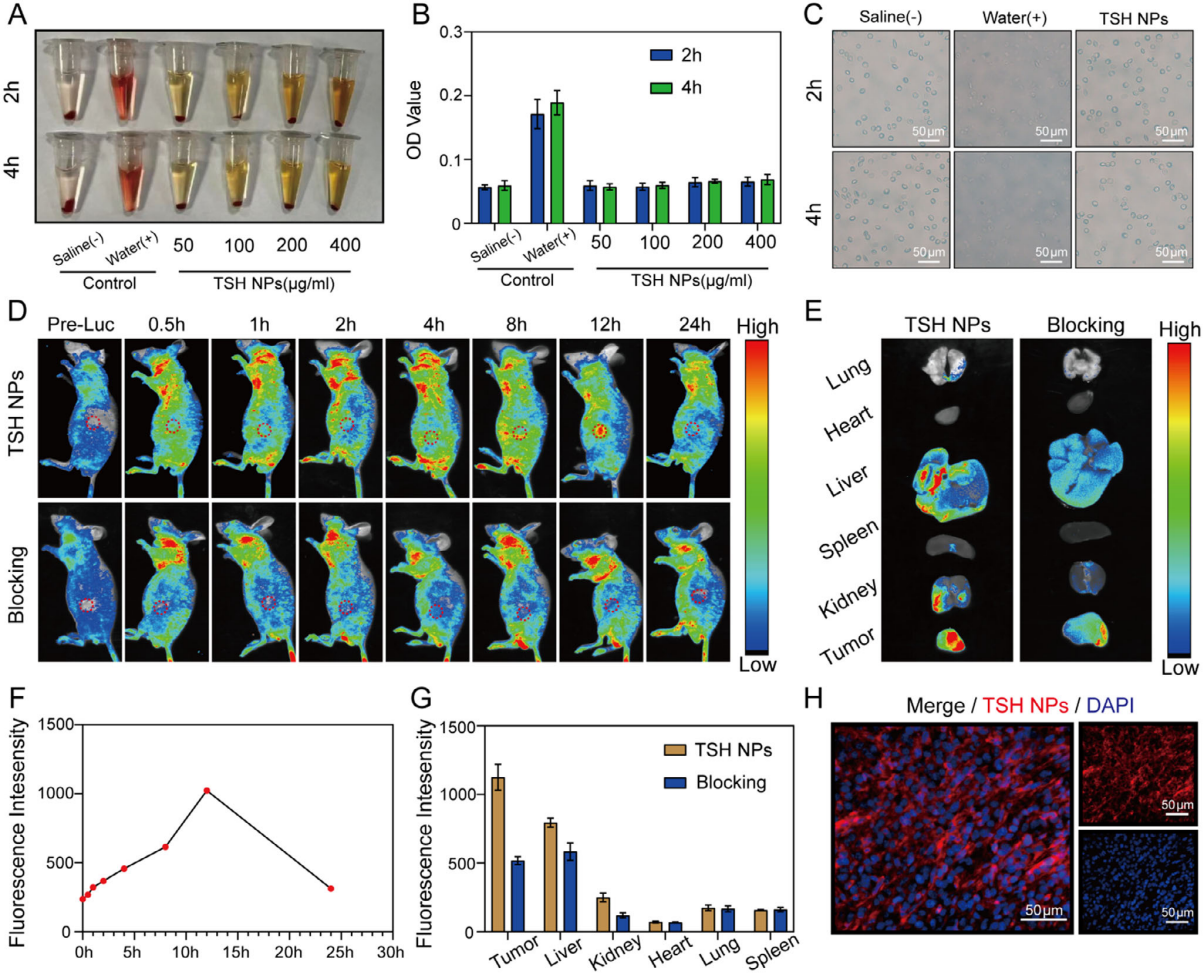
In vivo biosafety and tumor‐targeting capability of TSH NPs. (A) Representative bright‐field images of mouse red blood cells after incubation for 2 h and 4 h with saline, distilled water, or TSH NPs at different concentrations (50–400 µg/mL). (B) Absorbance values at 570 nm of the supernatants from the corresponding groups shown in (A). (C) Bright‐field images of red blood cell suspensions after incubation for 2 h and 4 h with saline, distilled water, or TSH NPs at a concentration of 400 µg/mL. (D) In vivo fluorescence imaging of mice following intravenous injection of TSH NPs or cilengitide pre‐treatment (blocking group) prior to TSH NPs injection. (E) Ex vivo fluorescence images of major organs and tumors collected 12 h after TSH NPs injection or after cilengitide pre‐treatment. (F) Time‐dependent changes in fluorescence intensity at tumor sites in mice following intravenous injection of TSH NPs. (G) Quantitative analysis of ex vivo fluorescence intensity in major organs and tumors at 12 h post‐injection, with or without cilengitide pre‐treatment. (H) Fluorescence microscopy images of excised tumor sections obtained 12 h post‐injection, showing red fluorescence from TSH NPs and blue DAPI‐stained nuclei.

Following the demonstration of potent in vitro anticancer efficacy, in vivo studies were conducted to further evaluate the tumor‐targeting capability and biodistribution of TSH NPs. Prior to systemic administration, the biocompatibility of TSH NPs at varying concentrations was assessed using hemocompatibility assays, with saline and distilled water serving as negative and positive controls, respectively. As shown in Figure 6A,B, co‐incubation of mouse erythrocytes with TSH NPs (50–400 µg/mL) for 2 and 4 h did not induce detectable hemolysis, whereas pronounced hemolysis was observed in the distilled water group. Subsequent smear analysis (Figure 6C) demonstrated that erythrocytes in both the TSH NPs and saline groups maintained intact morphology without observable structural abnormalities, whereas those in the distilled water group exhibited severe hemolysis and membrane disruption. These results indicate that TSH NPs exhibit excellent hemocompatibility across the tested concentration range, thereby validating their suitability for subsequent in vivo investigations. To evaluate the tumor‐targeting capability of TSH NPs under physiological conditions, a subcutaneous cholangiocarcinoma model was established in BALB/c nude mice using QBC939 cells. In vivo fluorescence imaging was performed to monitor nanoparticle accumulation at the tumor site over time. As shown in Figure 6D, in the non‐blocking group, TSH NPs gradually accumulated at the tumor site following tail vein injection, reaching peak fluorescence intensity at approximately 12 h post‐injection. The signal subsequently declined, with minimal fluorescence detectable after 24 h. In contrast, the blocking group, which was pretreated with cilengitide to inhibit receptor–ligand interactions, exhibited markedly reduced tumor‐associated fluorescence, suggesting that receptor‑mediated mechanisms contribute to the tumor accumulation of TSH NPs. However, it should be noted that the enhanced permeability and retention (EPR) effect, which is characteristic of nanoscale drug carriers in solid tumors, likely also contributes to passive accumulation at the tumor site. Fluorescence intensity profiling (Figure 6F) further demonstrated that TSH NPs were rapidly cleared from the body, suggesting a relatively short systemic half‐life that may help reduce exposure to healthy tissues and mitigate potential off‐target toxicity.

To further confirm biodistribution, ex vivo fluorescence imaging and quantitative analysis of major organs, including the heart, liver, spleen, lungs, kidneys, and tumors, were performed. As shown in Figure 6E,G, TSH NPs exhibited significantly higher fluorescence intensity in tumor tissues than in the blocking group, with comparatively lower nonspecific accumulation in other organs. Detectable fluorescence signals were observed in the liver and kidneys, indicating partial hepatic and renal clearance of TSH NPs. Tumor sections were further examined to assess intratumoral localization. As illustrated in Figure 6H, red fluorescence was predominantly distributed around DAPI‐labeled nuclei, confirming effective intratumoral delivery and cellular uptake of TSH NPs following intravenous administration.

To comprehensively evaluate systemic biosafety, therapeutic doses of TSH NPs were administered via tail vein injection, followed by assessment of serum biochemical indices and histopathological analysis of major organs. As shown in Figure S29, no significant differences were observed in serum levels of ALT, AST, LDH, BUN, CREA, or UA between the treatment and control groups (*p* > 0.05), indicating the absence of apparent hepatic or renal toxicity induced by TSH NPs or TPA NPs. Furthermore, no detectable pathological abnormalities were observed in major organs by H&E staining after short‑term treatment (Figure S30), and similarly, both serum biochemical parameters and H&E staining at 14 days post‑treatment (long‑term) revealed no significant abnormalities (Figure S31). Collectively, these findings demonstrate that TSH NPs possess favorable biocompatibility, effective tumor‐targeting capability, and minimal systemic toxicity in vivo, supporting their potential for further preclinical development and clinical translation.

### 2.7 In Vivo Anti‐Tumor Effects of TSH NPs and Attenuation of Adaptive Survival After PDT

**FIGURE 7:**
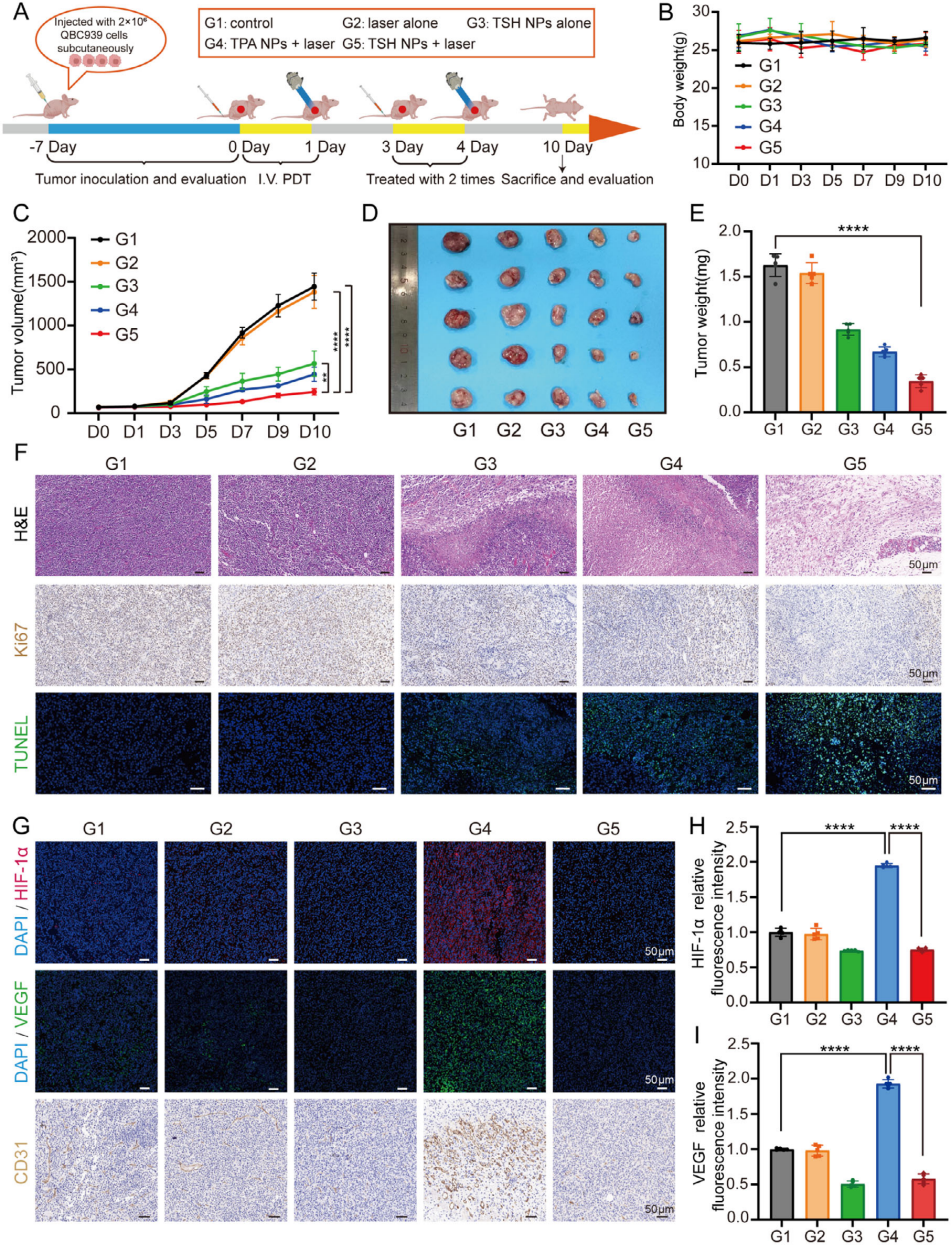
In vivo anti‐tumor effects of TSH NPs and attenuation of adaptive survival after PDT. (A) Schematic illustration of experimental grouping and treatment regimens in tumor‐bearing BALB/c nude mice. G1: intravenous (iv) injection of physiological saline; G2: iv injection of saline followed by blue‐light irradiation (490 nm, 960 mW/cm^2^, 10 min) after 12 h; G3: iv injection of TSH NPs (20 mg/kg) without light exposure; G4: iv injection of TPA NPs (20 mg/kg) followed by blue‐light irradiation after 12 h; G5: iv injection of TSH NPs (20 mg/kg) followed by blue‐light irradiation after 12 h. (B) Changes in body weight of mice during the treatment period. (C) Tumor growth curves showing tumor volume changes over time following different treatments. (D) Representative white‐light images of excised tumors collected at the end of the treatment period. (E) Quantitative comparison of tumor weights across different treatment groups. (F) Histological and immunohistochemical analyses of tumor tissues after treatment, including H&E staining, Ki‐67 immunohistochemistry, and TUNEL fluorescence staining. (G) Immunofluorescence staining of HIF‐1α and VEGF, and immunohistochemical staining of CD31 in excised tumor tissues following different treatments. (H–I) Quantitative analysis of HIF‐1α and VEGF fluorescence intensity in tumor sections across different treatment groups.

Encouraged by the effective tumor‐targeting performance observed in vivo and the potent cytotoxic effects demonstrated in vitro, we further evaluated the therapeutic efficacy of TSH NPs using a subcutaneous cholangiocarcinoma model established by inoculating BALB/c nude mice with QBC939 cells. When tumor volumes reached approximately 60 mm^3^, the mice were randomly divided into five groups (*n* = 5 per group). Based on the previously determined optimal intratumoral accumulation time of the nanomedicine, the treatment regimens were conducted as illustrated in Figure 7A: G1 (negative control group), tail vein injection of an equivalent volume of saline; G2 (light‐only group), tail vein injection of saline followed by tumor irradiation with blue light (490 nm, 960 mW/cm^2^, 10 min) after 12 h; G3 (chemotherapy‐only group), tail vein injection of TSH NPs (20 mg/kg) without subsequent light exposure; G4 (PDT group), tail vein injection of TPA NPs (20 mg/kg) followed by tumor irradiation with blue light (490 nm, 960 mW/cm^2^, 10 min) after 12 h; and G5 (combined treatment group), tail vein injection of TSH NPs (20 mg/kg) followed by tumor irradiation with blue light (490 nm, 960 mW/cm^2^, 10 min) after 12 h. The treatment regimen was repeated every 2 days following the initial administration.

Body weight and tumor volume were measured every other day to assess therapeutic efficacy. When tumor sizes in the negative control group approached the ethical limit for animal experimentation, all mice were euthanized, and tumor tissues were harvested for further analysis. As shown in Figure 7B, body weights remained relatively stable throughout the treatment period, with only minor weight loss observed in a small number of mice, indicating minimal systemic toxicity across the treatment groups. As illustrated in Figure 7C, tumor growth in both the negative control and light‐only groups accelerated markedly after day 5. In contrast, moderate tumor growth inhibition was observed in the chemotherapy and PDT groups. Notably, the combined treatment group exhibited a pronounced and sustained suppression of tumor progression. Quantitative analysis of tumor volume and weight on day 10 (Figure 7D,E) revealed that the tumor inhibition rate in the combined treatment group was 2.6‐fold higher than that in the chemotherapy group and 1.9‐fold higher than that in the PDT group. This superior antitumor efficacy is likely attributable to a synergistic therapeutic effect, whereby HCPT not only exerts direct cytotoxic activity but also mitigates the hypoxia‐induced signaling cascades triggered by PDT, thereby amplifying the overall therapeutic outcome. To further substantiate this hypothesis, H&E staining and immunohistochemical analyses were performed on excised tumor tissues. As shown in Figures 7F and S32, H&E staining demonstrated that the combination treatment group exhibited the most pronounced cytotoxic effects, as evidenced by extensive nuclear disruption and widespread cellular damage. In contrast, the chemotherapy‐only and PDT‐only groups displayed moderate levels of apoptosis and necrosis, whereas the control and light‐only groups largely preserved intact tumor cell morphology and tissue architecture.

Furthermore, immunohistochemical analysis of the proliferation marker Ki‐67 revealed that the combination treatment group achieved the greatest suppression of tumor cell proliferation. In addition, TUNEL staining further confirmed that the combination treatment induced more extensive apoptosis than the other treatment groups, thereby contributing to effective tumor reduction. CD31, a well‐established endothelial cell marker commonly used to evaluate microvessel density in tumor tissues, is closely associated with tumor oxygen supply, hypoxic microenvironment formation, and tumor cell adaptation. As shown in Figures 7G–I and S33, treatment with PDT alone resulted in a marked upregulation of HIF‐1α, VEGF, and CD31 expression compared with the negative control group. This observation is consistent with adaptive survival associated with rapid oxygen depletion during treatment. Such oxygen consumption exacerbates intratumoral hypoxia, thereby activating the HIF‐1α signaling pathway. The subsequent upregulation of pro‐angiogenic factors, including VEGF and CD31, facilitates neovascularization and enhanced tumor cell survival following PDT‐induced cytotoxicity, while also promoting tumor invasion and metastasis.

In contrast, both the chemotherapy‐only and combination treatment groups exhibited significantly reduced expression levels of HIF‐1α, VEGF, and CD31. These observations raise the possibility that HCPT may alleviate PDT‐induced hypoxia and suppress downstream pro‐survival and pro‐angiogenic signaling. Notably, the combination treatment group showed the most pronounced inhibition of these markers, suggesting that HCPT not only provides direct chemotherapeutic cytotoxicity but also is associated with reduced adaptive survival by interfering with hypoxia‐driven pathways. This may help to mitigate hypoxia‐associated adaptive survival and reduce the invasive and metastatic potential of QBC939 cells, which could explain the improved in vivo antitumor effects observed with the combination regimen.

## 3 Conclusion

In this study, a redox‐responsive prodrug nanoassembly was successfully developed to achieve targeted delivery to QBC939 cells. Comprehensive in vitro and in vivo investigations demonstrated that TSH NPs enabled efficient intracellular uptake via ligand–receptor‐mediated endocytosis, thereby facilitating the intracellular release of TPA to induce a potent PDT response. Concurrently, the co‐delivered HCPT not only exerted chemotherapeutic cytotoxicity but also was associated with attenuation of the PDT‐induced hypoxic tumor microenvironment and reduced activation of the HIF‐1α/VEGF signaling axis, contributing to reduced residual tumor viability. This residual viability reflects the acute adaptive survival that emerges when tumor cells surviving sublethal PDT mount hypoxia‐associated HIF‐1α/VEGF signaling and intracellular antioxidant defense responses, a phenomenon operationally defined as “PDT resistance” in the present study. As a major obstacle limiting the long‐term efficacy of PDT in eCCA, this adaptive survival phenotype represents a critical unmet clinical need and the central challenge that the present combination strategy was designed to address. Notably, this work represents an innovative exploration of the synergistic therapeutic mechanism between AIE photosensitizer‐mediated PDT and HCPT in the context of eCCA cells, offering a strategy to address this challenge. The dual‐targeting strategy and complementary actions observed here may help reduce tumor recurrence linked to PDT or chemotherapy resistance. Moreover, this combination strategy highlights the therapeutic potential of PDT‐centered multimodal regimens for the treatment of eCCA patients, while also offering the advantage of reduced systemic toxicity compared with HCPT monotherapy. Nevertheless, these in vivo findings were obtained using a subcutaneous xenograft model, which does not fully capture the native tumor microenvironment or metastatic progression of eCCA. Future studies using orthotopic or patient‐derived xenograft models, those exploring responsive and immunostimulatory nanocarriers [41, 42], are needed to further validate the translational potential. Overall, these findings provide a rationale that requires additional validation and serve as an initial preclinical foundation for future therapeutic design.

## Author Contributions

**Yong Qu**, **Tao Peng**, and **Yating Zhang** conceived the study, designed the experiments, performed cell and the animal studies, carried out staining and imaging, analyzed the data, and wrote the original draft. (These authors contributed equally to this work.). **Juanmei Cao**, **Birong Wang**, and **Chuxing Chai** performed cellular assays, prepared reagents and samples, curated data, and validated results. **Zhiyuan Gao**, **Yiting Xu**, **Yifan Jin**, and **Yuqing Wang** assisted with experiments, created figures and visualizations. **Zhuoxia Li**, **Xu Liu**, **Tianqi Zhao**, and **Yufan Chen** conducted experiments, optimized protocols, and helped validate findings. **Weimin Lv**, **Di Lu**, and **Changzheng Huang** supervised project implementation. **Dan Ding**, **Jinxiang Zhang**, and **Min Li** conceived the project, secured funding, supervised the research, and reviewed and edited the manuscript.

## Funding

This work was supported by the National Natural Science Foundation of China (82172754), Yangtze University Health Science Center, Yangtze University Medical Innovation Fund (grant number 2022MIF07), Doctoral Scientific Research Fund of The First Affiliated Hospital of Yangtze University (grant number 2022DIF05), the Joint Fund Project of Hubei Provincial Natural Science Foundation (2026AFC0601), and the Hebei Natural Science Foundation (B2025110067).

## Ethics Statement

All animal experiments were approved by the Ethics Committee of Tongji Medical College, Huazhong University of Science and Technology (Wuhan, China) ([2023] IACUC Number: 4359) and were performed in compliance with the institutional guidelines for the care and use of laboratory animals.

## Conflicts of Interest

The authors declare no conflicts of interest.

## Supporting information




**Supporting File**: adhm71599‐sup‐0001‐SuppMat.docx.

## Data Availability

The data that support the findings of this study are available on request from the corresponding author. The bioinformatics analysis of HIF1A expression and prognosis in cholangiocarcinoma was based on publicly available data from The Cancer Genome Atlas (TCGA‐CHOL; https://portal.gdc.cancer.gov/projects/TCGA‐CHOL) and the Genotype‐Tissue Expression project (GTEx; dbGaP accession phs000424), accessed via the GEPIA2 platform (http://gepia2.cancer‐pku.cn).
