# Supplementary material for: Overcoming Photodynamic Therapy Resistance: GSH‐Responsive AIE Photosensitizer‐Hydroxycamptothecin Nanoliposomes for Extrahepatic Cholangiocarcinoma
Source: Adv Healthc Mater. 2026 Aug 13;15(35):e71599. doi: 10.1002/adhm.71599 (PMC13586644; doi:10.1002/adhm.71599)
Supplement: Supplementary file 1 [file ADHM-15-0-s001.docx]

***Supporting Information***

**[Overcoming](https://www.thno.org/v15p7090.htm) Photodynamic Therapy Resistance: GSH-responsive AIE Photosensitizer-Hydroxycamptothecin Nanoliposomes for Extrahepatic Cholangiocarcinoma**

*Yong Qu ^a,b,1^, Tao Peng ^c,1^, Yating Zhang ^d,1^, Juanmei Cao ^e^, Birong Wang ^f^, Chuxing Chai ^a^, Zhiyuan Gao ^g^,Yiting Xu ^h^, Yifan Jin ^i^, Yuqing Wang ^j^, Zhuoxia Li ^a^, Xu Liu ^a^, Tianqi Zhao ^k^, Yufan Chen ^k^, Weimin Lv ^a^, Di Lu ^k^, Changzheng Huang ^k^, Dan Ding ^g,^*, Jinxiang Zhang ^b,^*, Min Li ^a,^**

^a^ Department of Hepatobiliary Surgery, Union Hospital, Tongji Medical College, Huazhong University of Science and Technology, Wuhan 430022, China.

^b^ Department of Emergency Surgery, Union Hospital, Tongji Medical College, Huazhong University of Science and Technology, Wuhan 430022, China.

^c^ Department of Hepatobiliary Surgery, The First Affiliated Hospital of Yangtze University, The First People’s Hospital of Jingzhou, Jingzhou 434007, China.

^d^ Department of Hepatobiliary Surgery, The First Affiliated Hospital of USTC, Division of Life Sciences and Medicine, University of Science and Technology of China, Hefei 230001, China.

^e^ Department of Dermatology, The First Affiliated Hospital of Shihezi University, Shihezi 832008, China.

^f^ Department of Thyroid Gland and Breast Surgery, Wuhan Fourth Hospital, Wuhan 430030, China.

^g^ Frontiers Science Center for Cell Responses, State Key Laboratory of Medicinal Chemical Biology, Key Laboratory of Bioactive Materials, Ministry of Education, and College of Life Sciences, Nankai University, Tianjin 300071, China.

^h^ Central Laboratory, Union Hospital, Tongji Medical College, Huazhong University of Science and Technology, Wuhan 430022, China.

^i^ Hospital for Skin Diseases,Institute of Dermatology,Chinese Academy of Medical Sciences&Peking Union Medical College, Nanjing 210042, China.

^j^ Department of Clinical Nutrition, Union Hospital, Tongji Medical College, Huazhong University of Science and Technology, Wuhan 430022, China.

^k^ Department of Dermatology, Union Hospital, Tongji Medical College, Huazhong University of Science and Technology, Wuhan 430022, China.

* Corresponding authors.

*E*-mail addresses: [liminmed@hust.edu.cn (](mailto:liminmed@hust.edu.cn(Li)M. Li), [zhangjinxiang@hust.edu.cn](mailto:zhangjinxiang@hust.edu.cn) (J. Zhang),

[dingd@nankai.edu.cn](mailto:dingd@nankai.edu.cn) (D. Ding).

^1^ These authors contributed equally to this work.

1. ***Materials and Methods***

***Materials and Equipments***

Extrahepatic cholangiocarcinoma cell line QBC939 and the normal human umbilical vein endothelial cell (HUVEC) were obtained from the Cell Bank of the Chinese Academy of Sciences (Shanghai, China). Fetal bovine serum (FBS), Dulbecco's modified Eagle's medium (DMEM), RPMI 1640 medium were obtained from Gibco (Grand Island, USA). 2',7'-dichlorofluorescin diacetate (DCFH-DA) and JC-10 were obtained from Hycezmbio (Wuhan, China). Lyso Tracker Green (LTG) was purchased from Thermo Fisher (Waltham, MA). Cell counting Kit-8 (CCK8) was purchased from Dojindo (Kumamoto, Japan). Actin-Tracker Green (F-actin), Triton X-100, Hoechst 33342, Annexin V-FITC/PI apoptosis detection kit, TUNEL apoptosis assay kit, Glutathione assay kit, nonfat powdered milk were purchased from Beyotime (Jiangsu, China). ECL substrate, RIPA lysis buffer, 4% paraformaldehyde, 2.5% glutaraldehyde buffer and DMSO were bought from Biosharp (Hefei, China). Calcein-AM/PI dual staining kit was purchased from Solarbio (Beijing, China). Anti-cleaved caspase-3 antibody was purchased from Cell Signaling Technology (Danvers, MA, USA). HIF-1α antibody, VEGF antibody and Goat Anti-Rabbit IgG antibody were obtained from Abcam (Cambridge, MA). BAK antibody, Cleaved Caspase 9 antibody, Cleaved PARP-1 antibody, Top I antibody, Tublin antibody, GAPDH antibody, Ki67 antibody and CD31 antibody, HRP-labeled anti-rabbit secondary antibody were bought from Proteintech (Wuhan, China). DAPI was bought from Invitrogen (Carlsbad, CA, USA). General Protease Inhibitor Cocktail and General Phosphatase Inhibitor Cocktail were purchased from Absin (Shanghai, China). BCA protein assay kit was bought from Boster (Wuhan, China). RIPA, TBS and PBS were bought from Servicebio Technology Co. Ltd (Wuhan, China). 5 × sample loading buffer, protein ladder and 12.5% gel kit were purchased from Epizyme (Shanghai, China). PVDF transfer membrane was bought from ThermoFisher (Waltham, MA, USA). All chemical agents were purchased from Aladdin Ltd unless otherwise noted. DSPE-PEG_2000_ and DSPE-PEG_2000_-cRGD were obtained from Xian rui’xi Biological Technology Co, Ltd.

UV-vis absorption spectra were measured using a Shimadzu UV-2600 spectrophotometer. Photoluminescence spectra were recorded using a Horiba Fluoromax-4 spectrofluorometer. Size and surface potential were measured by dynamic light scattering (DLS). Cells were imaged by confocal laser scanning microscope (CLSM, Nikon Corporation, Japan). Intracellular fluorescence intensity was determined by flow cytometry. Cell fluorescence was observed by fluorescence microscope. SDS-PAGE and western blots were performed with Wet/Tank Blotting Systems and were analyzed with ChemiDoc imaging system. In vivo fluorescence images were recorded by the Bruker In-Vivo FX PRO. Bruker AVANCE III 400MHz (Germany) was used to record 1H NMR of all chemical products. Bruker Solarix scimax MRMS (Germany) was used to obtain the HR-MS spectrum. The sample morphologies of TSH NPs were imaged on Cryogenic transmission electron microscopy (cryo-TEM; Talos L120C G2, Czech). Genesys 50 (Thermo, USA) was used to measure the UV–vis absorption spectrum. FS5 (Edinburgh, UK) was employed to measure the fluorescence spectrum. The sizes were measured with Nanoparticle Size Analyzer (Nano-ZS, UK).

***Synthesis of TSH***

The **Compound 1** synthesized according to the literature (300 mg, 0.58 mmol)^[35]^, 2-hydroxyethyl disulfide (265 mg, 1.73 mmol)，N-(3-dimethylaminopropyl)-N'-ethylcarbodiimide hydrochloride (EDCI, 275 mg, 1.44 mmol), and 4-dimethylaminopyridine (DMAP, 7 mg, 0.06 mmol) were dissolved in anhydrous dichloromethane. The air in flask was then repeatedly discharged and filled with nitrogen several times using the schlenk line. The flask was sealed and the reaction was stirred at room temperature for 24 h. Upon completion, the reaction mixture was diluted with dichloromethane and washed with saturated NaCl solution. And then, the aqueous phase was repeatedly extracted with dichloromethane, and the organic phase was separated and collected using separatory funnel. The organic phase was dried with anhydrous Na_2_SO_4_. On a rotary evaporator, the organic phase was concentrated and then purified by silica gel column chromatography (petroleum ether/ethyl acetate=2:1) to give **Compound 2** (63.1% yield). ^1^H NMR (400 MHz, DMSO-d_6_): δ 7.95 (s, 1H), 7.86 (d, J = 6.9 Hz, 2H), 7.72 (t, J = 4.0 Hz, 4H), 7.35 (t, J = 6.1 Hz, 4H), 7.16 – 7.05 (m, 6H), 7.03 (d, J = 6.8 Hz, 2H), 4.88 (s, 2H), 4.38 (s, 2H), 3.62 (s, 2H), 2.98 (s, 2H), 2.80 (s, 2H).

The **Compound 2** (240 mg, 0.36 mmol), 4-nitrophenyl chloroformate (108 mg, 0.55 mmol), DMAP (2.5 mg, 0.02 mmol) were added into anhydrous dichloromethane. N,N-Diisopropylethylamine (DIEA, 93 mg, 0.73 mmol) was added dropwise under nitrogen atmosphere at 0 °C. The reaction mixture was stirred at room temperature for 1 hour and monitored by Thin Layer Chromatography (TLC). Upon completion, the reaction mixture was diluted with dichloromethane and washed with saturated NaCl solution. And then, the aqueous phase was repeatedly extracted with dichloromethane, and the organic phase was separated and collected using separatory funnel. The organic phase was dried with anhydrous Na_2_SO_4_. On a rotary evaporator, the organic phase was concentrated, and then purified via column chromatography on silica gel (petroleum ether/ethyl acetate=10:1) to give **Compound 3** (61.2% yield). ^1^H NMR (400 MHz, Chloroform-*d*): δ 8.35 (d, *J* = 9.2 Hz, 2H), 8.27 (d, *J* = 9.2 Hz, 2H), 7.81 (s, 1H), 7.70 (d, *J* = 8.4 Hz, 2H), 7.56 (d, *J* = 8.4 Hz, 2H), 7.51 (d, *J* = 4.4 Hz, 2H), 7.39 (d, *J* = 9.1 Hz, 2H), 7.29 (t, *J* = 7.9 Hz, 4H), 7.17 – 7.14 (m, 4H), 7.07 (t, *J* = 7.3 Hz, 2H), 4.91 (s, 2H), 4.55 (t, *J* = 6.5 Hz, 2H), 4.48 (t, *J* = 6.5 Hz, 2H), 3.03 (t, *J* = 6.5 Hz, 2H), 2.98 (t, *J* = 6.5 Hz, 2H).

The **Compound 3** (179 mg, 0.22 mmol) and 10-Hydroxycamptothecin (121 mg, 0.33 mmol) were added into anhydrous dimethylformamide. Triethylamine (66 mg, 0.66 mmol) was added dropwise under nitrogen atmosphere at 0 °C. The reaction mixture was stirred at room temperature for 24 hours and monitored by TLC. Upon completion, the reaction mixture was diluted with ethyl acetate and washed with saturated NaCl solution. The organic phase was separated and then the residue in the aqueous phase was repeatedly extracted with ethyl acetate, and the organic phase was collected and dried with anhydrous Na_2_SO_4_. On a rotary evaporator, the organic phase was concentrated, and then purified via column chromatography on silica gel (dichloromethane/ methanol=20:1) to give TSH (62.6% yield). ^1^H NMR (400 MHz, Chloroform-*d*): δ 8.38 (s, 1H), 8.25 (d, *J* = 9.1 Hz, 1H), 7.81 (s, 1H), 7.75 (d, *J* = 2.1 Hz, 1H), 7.74 – 7.64 (m, 4H), 7.51 (t, *J* = 6.5 Hz, 4H), 7.34 – 7.29 (m, 4H), 7.18 – 7.04 (m, 8H), 5.73 (d, *J* = 16.3 Hz, 1H), 5.39 – 5.21 (m, 3H), 4.90 (s, 2H), 4.58 (t, *J* = 6.2 Hz, 2H), 4.50 (t, *J* = 5.1 Hz, 2H), 3.08 (t, *J* = 5.1 Hz, 2H), 3.01 (t, *J* = 5.1 Hz, 2H), 2.34 (d, *J* = 2.1 Hz, 2H), 2.02 (s, 2H), 1.04 (t, *J* = 6.1 Hz, 3H), 0.94 – 0.80 (m, 2H). HRMS (ESI) *m/z* [M+H]^+^ calculated for C_55_H_45_N_4_O_10_S_4_: 1049.2019, found: 1049.1996.

***Detection of ROS Production with DCFH in Solution***

Detection of ROS production with DCFH in solution Compound 2',7'-dichlorodihydrofluorescein (DCFH) was used as indicator for detection of ROS in solution. When ROS is generated in the system, DCFH will be oxidized, leading to an increase in fluorescence at 524 nm. Photosensitizers (10 µM) were dispersed in 2 mL PBS containing 50 µM DCFH. This mixture was placed in a cuvette and irradiated with white light (0.2 W/cm^2^). The fluorescence intensity from 480 nm to 600 nm was recorded by the fluorescence spectrometer.

***Preparation of TPA NPs and TSH NPs***

1 mg of TSH, 2.1 mg of DSPE-PEG_2000_ and 0.7 mg of DSPE-PEG_2000_-RGD are weighted and dissolved in 1 mL THF. Then, the mixture is added dropwise into DI water under ultrasound sonication, which is followed by nitrogen blowing to remove THF and filtration using a 0.45 μm syringe filter and concentrated by ultrafiltration.

The encapsulation efficiency (EE) and drug loading capacity (DLC) of TPA and TSH were determined using UV spectrophotometer and calculated according to their absorption coefficients.

EE = (Weight of TSH in TSH NPs)/(Weight of the feeding TSH)×100%

DLC = (Weight of TSH in TSH NPs)/(Weight of TSH NPs)×100%

The procedure of TPA NPs is the same as that of TSH NPs.

***Release of HCPT from TSH NPs***

Drug release curve in vitro of TSH NPs was determined as follows: TSH NPs solution (1 mL) was placed in a dialysis bag (molecular cut off: 10 kDa) and immersed the dialysis bag in PBS solutions (4 mL) or PBS solution containing 10 mM GSH. In 37 °C constant temperature water bath, buffer solution (4 mL) was taken out at different time points and fresh buffer solution (4 mL) was added again. Then add DMSO (1 mL) to the buffer solution (1 mL) taken out to get the mixed solvent of water and DMSO. Using the fluorescent spectrometer to measure the fluorescence intensity of the solution at the wavelength of 587 nm, according to the standard curve to calculate the cumulative release amount of HCPT.

***Cell Culture***

QBC939 cells were maintained in Roswell Park Memorial Institute (RPMI)-1640 medium (Gibco). The media were supplemented with 10% fetal bovine serum (FBS, Gibco) and 1% penicillin‒streptomycin (P/S). The HUVEC cells were cultured in high-glucose Dulbecco's modified Eagle's medium (DMEM, Gibco). The cells were obtained from Shanghai Cell Bank of Chinese Academy of Sciences (Shanghai, China) and were cultured at 37 ℃ in a humidified incubator with 5% CO2.

***The Absorption of TSH NPs in QBC939 Cells***

Confocal Laser Scanning Microscope (CLSM, Nikon Corporation, Japan) and flow cytometry (FCM, BD, CA, USA) were applied as means to monitor the absorption of TSH NPs in QBC939 cells over time and at different concentrations. Briefly, QBC939 cells were seeded into slide chambers for overnight growing to 70% density, and then incubated with 30 μM TSH NPs for 2, 4 and 6 h, separately. Then, the cells were fixed with 4% formaldehyde for 10 min and washed 3 times with PBS containing 0.1% Triton X-100 for 5 min each time. Immediately thereafter, the fixed QBC939 cells were successively labelled with 10 μg/mL Actin-Tracker in the dark for 40 min and stained with DAPI for 5 min. Between each step, cells were washed 3 times using PBS for 5 min each time. Then the cells were imaged using a CLSM. For TSH NPs: λex = 488 nm and band-pass filter λ = 552–617 nm. For DAPI: λex = 405 nm and band-pass filter λ = 425–475 nm. For F-actin: λex = 546 nm and band-pass filter λ =552–617nm. Following the same methodology as above, QBC939 cells were incubated with 15, 30 and 60 μM TSH NPs for 6 h, separately. Similarly, the cells were fixed with 4% formaldehyde for 10 min and washed 3 times with PBS containing 0.1% Triton X-100 for 5 minutes each time. Finally the groups were fixed with DAPI and observed under CLSM. For TSH NPs: λex = 488 nm and band-pass filter λ = 552–617 nm. For DAPI:λex = 405 nm and band-pass filter λ = 425–475 nm.

***Quantitative Analysis of Cellular Uptake by Flow Cytometry***

QBC939 cells were inoculated into 6-well plates and used for experiments when the density of cells grew to 80%. Cells were first incubated with 15 μM, 30 μM, and 60 μM TSH NPs for 6 h and then washed three times with PBS. Immediately after that, they were digested with trypsin for 2 min and then placed in a flow-through tube. The cells were washed again with PBS for three times and then prepared for examination. In the same way as above, the cells were incubated with 30 μM TSH NPs for 2 h, 4 h and 6 h respectively and then placed in a flow-through tube as described above for examination. The fluorescence intensity of the cells was determined by a FCM. The cellular uptake analysis was performed directly using flow cytometry.

***Cell Targeting and Co-localisation Distribution Experiments***

For the cell targeting experiments, cilengitide was used as the integrin ανβ3 inhibitor. Cilengitide (0.1 μg/mL) was first incubated with QBC939 cells for 6 h before TSH NPs were added. Vascular endothelial HUVEC cells were used as normal controls. QBC939 cells, blocker-pretreated QBC939 cells, and HUVEC cells were inoculated into 35-mm confocal dishes and then co-incubated with 30 μM TSH NPs for 6 h.Then, the cells were fixed with 4% formaldehyde for 10 min and washed 3 times with PBS containing 0.1% Triton X-100 for 5 min each time. Immediately thereafter, the fixed cells were successively labelled with 10 μg/mL Actin-Tracker Red in the dark for 40 min. Then the cells were imaged using a CLSM. For TSH NPs: λex = 488 nm and band-pass filter λ = 552–617 nm. For DAPI: λex = 405 nm and band-pass filter λ = 425–475 nm. For F-actin: λex = 546 nm and band-pass filter λ = 552–617 nm. Fluorescence quantification of each group was analysed separately using Image J for intracellular probe co-localisation experiments, QBC939 cells were incubated with TSH NPs for 6 h and stained with 1 mM Lysotracker Green. After washing three times with PBS, Hoechst 33342 was used to localize cell nuclei. Eventually, the cells were imaged by the above-mentioned confocal microscopy. For TSH NPs: λex = 488 nm and band-pass filter λ = 552–617 nm. For Hoechst 33342: λex = 405 nm and band-pass filter λ = 425–475 nm. For Lysotracker: λex =488 nm and band-pass filter λ = 500–530 nm.

***Intracellular ROS Detection***

We used a commercial DCFH-DA probe for the detection of intracellularly produced reactive oxygen species according to the instructions. Specifically, QBC939 cells were seeded into 35 mm culture plates and randomly divided into negative control group, rosup positive group, TSH NPs alone group, TSH NPs + Light group, and TPA NPs + Light group. Firstly, 30 μM TSH NPs, TPA NPs were co-incubated with QBC939 cells for 6 h. After washing the residual nanomedicines in the culture plates with PBS, all groups were incubated with 10 μM DCFH-DA at 37 °C for 30 min in the dark. Immediately after that, the culture plate was washed with PBS again for 3 times and then the TSH NPs + Light group, TPA NPs + Light group were exposed to light (300 mW/cm^2^) for 5 min, and the rosup-positive group was incubated with rosup for 20 min under dark conditions. Fluorescence from DCF was observed using fluorescence microscope (IX71, Olympus, Japan) after irradiation.

***In Vitro Cytotoxicity Assay***

Cell viability after different treatments was evaluated using CCK8 assays. Briefly, QBC939 cells were inoculated into 96-well plates and used for experiments when the density reached 70%. For the dark toxicity experiments with the photosensitiser TPA, different concentrations of TPA NPs (0, 15, 30, 60, 90 μM) were co-incubated with the cells under dark conditions at 37°C for 6 h. Subsequently, the cells were incubated with 100 mL of fresh medium (including 10 mL of CCK8 solution per well) for 2 h according to its instructions. For concentration killing gradient experiments, different concentrations of TSH NPs (0, 10, 20, 40, 60 μM) were co-incubated with QBC939 at 37°C in the dark for 6 h. After washing with PBS and replacing with fresh medium, the incubation was continued to 24 h. The old medium was then removed, and the cells in each well were incubated with 100 mL of fresh medium (including 10 mL of CCK8 solution per well) for 2 h for testing. Cells were then incubated with 100 mL of fresh medium (including 10 mL of CCK8 solution per well) for 2 h. In the same way as above, QBC939 cells were co-incubated with 30 μM TSH NPs for 6 h and then washed with PBS. Subsequently exposed to blue light (300 mW/cm^2^ ) by following the time gradient (0/2/5/8 min). After an interval of up to 24 h, the old medium was similarly removed and the cells in each well were incubated with with 100 mL of fresh medium (including 10 mL of CCK8 solution per well) for 2 h to be measured. Finally, to evaluate the synergistic therapeutic effects of photodynamic and chemotherapy, QBC939 cells were co-incubated with 30 μM TSH NPs and 30 μM TPA NPs for 6 h and then exposed to blue light (300 mW/cm^2^) for 5 min. QBC939 cells were co-incubated with 30 μM TSH NPs at 37 ℃ in the dark for 6 h as the chemotherapy experimental group, and normal-growth QBC939 cells were used as the negative control. The remaining experimental treatments were the same as this experimental condition and grouping. The above treatments were replaced with fresh medium and incubated for another 24 h before being tested according to the CCK8 experimental method described above. The absorbance of solution at 450 nm was detected using a Multiskan GO Microplate Spectrophotometer (Thermo Scientific, USA).

***Calcein-AM/Propidium Iodide (PI) Dual Fluorescence Staining***

Calcein-AM/PI dual fluorescent assay was used to differentiate the live and dead cells according to the protocol. After different treatments, the mixture of calcein-AM (2 μM) and PI (5 μM) was added to the cells and further incubated for 20 min at 37 °C. Fluorescent images were visualized under fluorescence microscope (IX71, Olympus, Japan). Randomized microscopic fields were selected to count the percentage of the live (calcein-positive) and dead (PI-positive) cells.

***Mitochondrial Membrane Potential (ΔΨm) Measurement***

The ΔΨm of cells was detected by JC-10 mitochondrial membrane potential probe in accordance with the manufacturer’s protocol. Briefly, after different treatments, cells were incubated with 2 μg/mL of JC-10 for 20 min at 37 °C. After washing twice with PBS, the cells were observed using fluorescence microscopy.

***Flow Cytometry Analysis of Apoptosis and Necrosis***

The proportion of apoptotic and necrotic cells were quantified using Annexin V-fluorescein isothiocyanate (FITC)/propidium iodide (PI) dual staining. Briefly, QBC939 cells were exposed to different treatments in the same way as above and further incubated for 24 h. The cells were then washed twice with PBS and harvested after digestion with trypsin. The cells were centrifuged and resuspended in 100 μL of binding buffer. They were then stained with 5 μL Annexin V-FITC and 10 μL PI for 15 min in the dark at room temperature, then analyzed using flow cytometry (BD, CA, USA) within 1 h.

***Detection of Intracellular GSH Levels***

Configure the relevant assay reagents according to the assay kit instructions. After grouping and treatment as described above, PBS washed QBC939 cells once, centrifuged to collect cells, and aspirated the supernatant. Then, adding 3 times the volume of cell sediment volume of protein removal reagent S solution, fully Vortex. The samples were then subjected to two rapid freeze-thaws using liquid nitrogen and a 37°C water bath. Immediately thereafter, the samples were left at 4°C or an ice bath for 5 min and centrifuged at 4°C for 10 minutes at 10,000 g. The supernatant was taken for total glutathione determination. Briefly, following the instructions, using a 96-well plate, samples or standards were added sequentially and mixed. Immediately after adding 150 µL of Total Glutathione Assay Working Solution, mixed well and incubated for 5 min at room temperature. After that, 50 µL of 0.5 mg/ml NADPH solution was added and mixed well. Immediately after the reaction, the absorbance of solution at A412nm was detected using a Multiskan GO Microplate Spectrophotometer (Thermo Scientific, USA).

***Immunofluorescence Staining for HIF-1α***

QBC939 cells were subjected to immunofluorescence staining immediately after the different treatments described above. The above cells were fixed with 4% formaldehyde at room temperature for 20 min and blocked with 10% BSA/Triton X-100 in PBS for 1h. Thereafter, the cells were incubated with mouse monoclonal antibody HIF-1α antibody (1:50) at 4 ℃ overnight, washed twice, and then incubated with phycoerythrin PE coupled with anti-mouse secondary antibody (1:100; Invitrogen) at room temperature for 1 h. Finally the cells were fixed with DAPI and observed under Confocal Laser Scanning Microscope (CLSM, Nikon Corporation, Japan).

***Western Blotting***

Firstly, the QBC939 cellular proteins which were treated according to the above conditions were extracted. The treated cells were rinsed twice with pre-cooled PBS and pre-cooled lysate was added. Subsequently, the adherent cells were scraped with a cell scraper and the cells and lysate were gently transferred to the pre-cooled centrifuge tubes. Subsequently, the centrifuge tubes were placed in an ultrasonic crusher for full lysis and crushing, and then the centrifuge tubes were then subjected to a high speed centrifugation at 12,000 rpm for 20 min at 4℃. Gently aspirate the supernatant from the centrifuge tubes and transfer it to the new pre-cooled centrifuge tubes, the supernatant obtained from the above operation is the protein sample. The BCA method as the method for protein quantification. Briefly, based on the absorbance values of the protein samples measured above, the corresponding protein content could be found on the standard curve. Afterwards, the denatured protein samples (20 μg per lane) were separated by SDS-PAGE on a 10% gel, transferred to a polyvinylidene difluoride (PVDF) membrane, blocked with 5% skimmed milk for 1 h at room temperature, and then incubated overnight at 4 °C with the corresponding primary antibodies (HIF-1α, VEGF, BAK,Cleaved Caspase 3, Cleaved Caspase 9, Cleaved PARP-1, Top I, GAPDH, Tublin) overnight at 4 ℃. The following antibodies were used: anti-HIF-1α (Abcam, 1:1000), anti-VEGF (Abcam, 1:1000), anti-BAK (Proteintech, 1:1000), anti-Cleaved Caspase 3 (CST, 1:2000), anti-Cleaved Caspase 9 (Proteintech, 1:1000), anti-Cleaved PARP-1 (Proteintech, 1:1000), anti-Top I (Proteintech, 1:1000), anti-GAPDH (Proteintech, 1:1000), anti-Tublin (Proteintech, 1:2000). The membrane is washed three times with Tris buffered saline Tween-20 (TBST) and incubated with secondary antibody for 1 h at room temperature. Eventually, the bands of immunoreactive proteins on the membrane are detected using the Enhanced Chemiluminescence (ECL) detection system.

***Cell Scratching and Apoptosis Observation Experiment***

QBC939 cells in the logarithmic growth phase were trypsin-digested into single-cell suspensions and inoculated into 6-well culture plates. Subsequently, the cells were incubated in a 37°C, 5% CO2 incubator for 24 h, and then scratched vertically with a 200 microlitre tip when the cells were spread out. After rinsing the cells 3 times with PBS, the scratched cells were removed and serum-free medium was added. Following treatment in the above groupings, samples were taken at 0, 24 and 48h time points and photographed. Similarly, QBC939 cells in the logarithmic phase of growth were inoculated in petri dishes and treated in groups as described above, and then photographed by fluorescence microscopy (IX71, Olympus, Japan) at 0, 24, and 48 h.

***Single-cell Analysis for Cell type identification and Differential expression***

The data used in the above research come from the files in the Supplementary file of the GEO database (https://www.ncbi.nlm.nih.gov/geo/). Download the corresponding single-cell data in .h5 format and annotation results from TISCH. Use the R software MAESTRO and Seurat to process and analyze the single-cell data. Re-cluster the cells using the t-SNE method.

***Bioinformatics Analysis Using GEPIA2***

The mRNA expression levels of HIF1A in cholangiocarcinoma (CHOL) tumor tissues versus adjacent non-tumor tissues were analyzed using the Gene Expression Profiling Interactive Analysis 2 (GEPIA2) platform (http://gepia2.cancer-pku.cn), which integrates RNA-seq data from The Cancer Genome Atlas (TCGA) and Genotype-Tissue Expression (GTEx) projects. Differential expression was evaluated with default settings (|log_2_FC| > 1, P < 0.05). For survival analysis, CHOL patients were stratified into high and low HIF1A expression groups based on the median expression value. Overall survival curves were generated using the Kaplan-Meier method, and the log-rank test was applied to assess statistical significance. Hazard ratios (HR) and 95% confidence intervals (CI) were calculated using univariate Cox regression analysis within the GEPIA2 tool. No written consent has been obtained from the patients as there is no patient-identifiable data included in this study.

***mRNA Sequencing Experimental Method***

RNA Isolation and Library Preparation: Total RNA was extracted using the TRIzol reagent (Invitrogen, CA, USA) according to the manufacturer’s protocol. RNA purity and quantification were evaluated using the NanoDrop 2000 spectrophotometer (Thermo Scientific, USA). RNA integrity was assessed using the Agilent 2100 Bioanalyzer (Agilent Technologies, Santa Clara, CA, USA). Then the libraries were constructed using VAHTS Universal V10 RNA-seq Library Prep Kit (Premixed Version) according to the manufacturer’s instructions. The transcriptome sequencing and analysis were conducted by OE Biotech Co., Ltd. (Shanghai, China).

***mRNA Sequencing Analysis Process***

RNA Sequencing and Differentially Expressed Genes Analysis：The libraries were sequenced on an Illumina Novaseq X Plus platform and 150 bp paired-end reads were generated. Raw reads of fastqformat were firstly processed using fastp and the low quality reads were removed to obtain the clean reads. The clean reads were mapped to the reference genome using HISAT2 FPKM of each gene was calculated and the read counts of each gene were obtained by HTSeq-count. PCA analysis were performed using R (v 3.2.0) to evaluate the biological duplication of samples. Differential expression analysis was performed using the DESeq2 foldchange > 1.5 or foldchange < 0.67 was set as the threshold for significantly differential expression gene (DEGs). Hierarchical cluster analysis of DEGs was performed using R (v 3.2.0) to demonstrate the expression pattern of genes in different groups and samples. Based on the hypergeometric distribution, GO, KEGG pathway, Reactome and WikiPathways enrichment analysis of DEGs were performed to screen the significant enriched term using R (v 3.2.0), respectively. R (v 3.2.0) was used to draw the column diagram, the chord diagram and bubble diagram of the significant enrichment term.

***Animal Tumor Model***

4-week-old male BALB/c nude mice were purchased from SiPeiFu (SPF) Biotechnology Co., Ltd. (Beijing, China) and housed under specific pathogenfree (SPF) conditions at Tongji Medical College Animal Experimentation, and the experiments were performed in compliance with the guidelines established by the Ethics Committee of Tongji Medical College, HUST, Wuhan, China ([2023] IACUC Number: 4359). To establish tumor models, 2 × 10^6^ QBC939 cells were inoculated subcutaneously into the upper right thigh of BALB/c nude mice. Tumor growth was measured using a caliper, and tumor volume was calculated using the formula: volume = ((tumor length) × (tumor width)^2^)/2.

***In Vivo Fluorescence Imaging and Biodistribution Analysis***

Following the above steps, the nude mouse animal model was established and used for experiments when the tumour volume reached 60 mm^3^. Based on the body weight of the mice, TSH NPs were injected into the tail vein at 20 mg/kg. For the blocking experiment, cilengitide (100 μg) was administered to mice 30 min before the administration of TSH NPs. Real-time fluorescence imaging was performed using an In-Vivo FX PRO (BRUKER, Germany) for preset times. Subsequently, nude mice in the receptor-blocked and non-blocked groups were executed, and major organs (liver, kidney, spleen, heart, lung, brain and intestine) and tumours were collected. The fluorescence intensity of each organ and tumour was measured by In-Vivo FX PRO (BRUKER, Germany) in both groups (n = 3 per group). Tumors were fixed using 10% formalin for at least 24 h. DAPI was used to stain for the nuclei of cells. Fluorescence images were acquired using fluorescence microscope.

***Biosafety and Hemolysis Assay***

Briefly, the blood sample (1 mL) of BALB/c mice obtained from the orbital vein is diluted with 2 mL of PBS. Separate red blood cells (RBCs) from serum by centrifugation at 2000 rpm for 10 min. After washing 3 times, the RBCs are then diluted with 10 mL of PBS. The erythrocyte suspension (30 μL) is mixed with 120 μL of PBS (negative control), distilled water (positive control), and TSH NPs at various concentrations (50-400 μg/mL). The mixtures were incubated at 37 ℃ for 2 h and 4 h and centrifuged at 12000 rpm for 10 min. Hemolytic images were collected and the supernatant from each tube (100μL) was added to a 96-well plate and their absorbance at 570 nm was measured using a Multiskan GO microplate spectrophotometer (Thermo Scientific, USA). For the biosafety experiments, nude mice were subsequently divided into the negative control group, the TPA NPs injection group, the TSH NPs injection group. According to the grouping, saline, TPA NPs (20 mg/kg), TSH NPs (20 mg/kg) were injected into nude mice through the tail vein respectively. After the interval of 24 h, venous blood was taken from the orbital vein of nude mice in groups for biochemical tests, and mice were dissected for HE staining of heart, liver, spleen, lungs and kidneys to observe the presence of organ damage.

***Anti-tumour Effects of Photodynamic Combined Chemotherapy in Vivo***

Selected QBC939 cell-loaded BALB/c nude mice with uniform tumour size were used for in vivo PDT study. When the tumour volume reached approximately 60 mm^3^ , the mice were randomly divided into 5 groups (n = 5 each). 5 groups were treated as follows: (1) Group 1 (negative control) was injected with saline via tail vein only (2) Group 2 was injected with saline and then irradiated with laser light (490 nm, 960 mW/cm^2^ ) 12 h after the injection (3) Group 3 was injected with TSH NPs without laser light (4) Group 4 was injected with TPA NPs, followed by irradiation (490 nm, 960 mW/cm^2^) (5) Group 5 was injected with TSH NPs, followed by irradiation (490 nm, 960 mW/cm^2^). Each group was treated with the above drugs twice via tail vein on the first day and the third day, respectively, and the tumour volume and body weight of the mice were measured every other day. Finally, the mice were executed, and the tumour tissues and major organs of each group were collected for H&E staining. The proliferation, angiogenesis, apoptosis and hypoxia of the tumour tissues were studied by Ki-67 staining, CD31 staining, TUNEL staining and immunofluorescence staining with HIF-1α and VEGF.

***Statistical Analysis***

Quantitative fluorescence analysis was performed using image J, and statistical analysis and data analysis were performed using GraphPad Prism 9.5 software. Results are expressed as SD ± mean. p < 0.05 or p < 0.01 marked with ‘**’ or ‘****’ were considered significant. One-way ANOVA with Tukey’s multiple comparisons was used for multiple comparisons when more than two groups were compared, and two-tailed Student’s t-test was used for two-group comparisons. Bioinformatic analysis was performed using the OECloud tools at https://cloud.oebiotech.com/task/. The volcano map (or other graphics) was drawn based on the R (https://www.r-project.org/) on the OECloud platform ( <https://cloud.oebiotech.com/task/).> The mechanism diagrams in this paper were created by Figdraw(ID:SUITU33274, ID:USPYR08899, ID:YUUPY07702).

2. ***Supplementary Figures***

***Figure S1****.* ^1^H NMR spectrum of **Compound 2**.

***Figure S2****.* ^1^H NMR spectrum of **Compound 3**.

***Figure S3****.* ^1^H NMR spectrum of TSH.

***Figure S4****.* HRMS spectrum of compound TSH.

***Figure S5****.* The fluorescent spectra of TSH (10 μM) in DMSO/toluene mixtures with different toluene fractions.

***Figure S6****.* ROS generation by Ce6 (10 μM) under the irradiation of white light (0.2 W/cm^2^), using DCFH as the ROS probe.

***Figure S7****.* ROS generation by TSH (10 μM) under the irradiation of white light (0.2 W/cm^2^), using DCFH as the ROS probe.

***Figure S8****.* Representative dynamic light scattering result and transmission electron microscopy image of the TPA NPs.

***Figure S9****.* The UV/Vis absorption standard curves of TPA NPs.

.

***Figure S10****.* The fluorescence spectral changes of TSH (10 μM) with increasing GSH (10 mM) incubation time.

***Figure S11****.* ROS generation kinetics of TSH NPs with and without GSH under visible light irradiation (0.2 W/cm²), assessed by DCFH oxidation. Fluorescence intensity ratios (I/I_0_) of DCF at 524 nm were plotted against irradiation time, with I_0_ representing the baseline intensity prior to light exposure.

***Figure S12****.* Intracellular ROS generation in QBC939 cells following treatment with TSH NPs alone.

***Figure S13****.* Intracellular ROS generation in QBC939 cells following treatment with Rosup.

***Figure S14****.* Representative images illustrating morphological changes in QBC939 cells following different treatments: negative control, chemotherapy alone, TPA NPs + laser irradiation, and TSH NPs + laser irradiation at 0 h.

***Figure S15****.* Quantitative analysis of BAK protein expression levels under the different treatment conditions.

***Figure S16****.* Quantitative analysis of cleaved caspase-3 protein expression levels under the different treatment conditions.

***Figure S17****.* Quantitative analysis of cleaved caspase-9 protein expression levels under the different treatment conditions.

***Figure S18****.* Quantitative analysis of cleaved PARP-1 protein expression levels under the different treatment conditions.

***Figure S19****.* Quantitative analysis of Top I protein expression levels under the different treatment conditions.

***Figure S20****.* Expression distribution of *HIF1A* in bile duct cancer tissues and normal tissues.

***Figure S21****.* Gene expression distribution across the control (C1,C2,C3), TPA NPs + Light (A1,A2,A3), and TSH NPs + Light (S1,S2,S3) groups. Colors correspond to different FPKM value ranges. The horizontal axis indicates individual samples, and the vertical axis shows the count of protein-coding genes.

***Figure S22****.* Correlation coefficient plot of sequencing samples based on gene expression patterns in the control (C), TPA NPs + Light (A), and TSH NPs + Light (S) groups. The x- and y-axes represent sample names, and colour intensity corresponds to the correlation coefficient value.

***Figure S23****.* Principal component analysis (PCA) was performed to assess global transcriptomic differences among groups.

***Figure S24****.* Clustering analysis of sequencing samples based on gene expression patterns; Sample names are displayed on both axes, and colour intensity indicates the degree of similarity, with darker shades representing closer relationships.

***Figure S25****.* Gene Ontology (GO) enrichment analysis of differentially expressed genes between the control (C1-C3) and TPA NPs + Light (A1-A3) groups.

***Figure S26****.* Protein-protein interaction (PPI) network of differentially expressed genes between the control (C1-C3) and TPA NPs + Light (A1-A3) groups.

***Figure S27****.* Gene Ontology (GO) enrichment analysis of differentially expressed genes between the TSH NPs + Light (S1-S3) and TPA NPs + Light (A1-A3) groups.

***Figure S28****.* PPI network of differentially expressed genes between TSH NPs + Light (S1-S3) and TPA NPs + Light (A1-A3) groups.

***Figure S29****.* Serum biochemical parameters (ALT, AST, LDH, BUN, CREA, and UA) in mice 24 h after intravenous injection of saline, TPA NPs, or TSH NPs, indicating systemic biosafety.

***Figure S30****.* The **hematoxylin and eosin (H&E) staining of the heart, liver, lungs, spleen, kidneys, and tumor tissues** in mice 24 h after intravenous injection of TSH NPs.

***Figure S31****.* Serum biochemical parameters and H&E staining at 14 days post‑treatment. (A): changes in serum biochemical parameters after intravenous injection of saline, TPA NPs, or TSH NPs. (B): H&E staining sections of the heart, liver, spleen, lungs, and kidneys after intravenous injection of saline, TPA NPs, or TSH NPs.

***Figure S32****.* Quantitative analysis of Ki-67 immunohistochemical staining in tissue sections from treatment groups G1-G5.

***Figure S33****.* Quantitative analysis of CD31 immunohistochemical staining in tissue sections from treatment groups G1-G5.
